# Synthesis of zigzag- and fjord-edged nanographene with dual amplified spontaneous emission†

**DOI:** 10.1039/d2sc04208h

**Published:** 2022-10-18

**Authors:** Xiushang Xu, Gianluca Serra, Andrea Villa, Rafael Muñoz-Mármol, Serhii Vasylevskyi, Marcos Gadea, Andrea Lucotti, Zensen Lin, Pedro G. Boj, Ryota Kabe, Matteo Tommasini, María Á. Díaz-García, Francesco Scotognella, Giuseppe Maria Paternò, Akimitsu Narita

**Affiliations:** Organic and Carbon Nanomaterials Unit, Okinawa Institute of Science and Technology Graduate University 1919-1 Tancha, Onna-son, Kunigami-gun Okinawa 904-0495 Japan akimitsu.narita@oist.jp; Max Planck Institute for Polymer Research Ackermannweg 10 55128 Mainz Germany; Dipartimento di Chimica, Materiali e Ingegneria Chimica ‘G. Natta’, Politecnico di Milano Piazza Leonardo da Vinci 32 20133 Milano Italy; Physics Department, Politecnico di Milano Piazza L. da Vinci 32 Milano 20133 Italy giuseppemaria.paterno@polimi.it; Engineering Section, Research Support Division, Okinawa Institute of Science and Technology Graduate University 1919-1 Tancha, Onna-son, Kunigami-gun Okinawa 904-0495 Japan; Departamento de Física Aplicada and Instituto Universitario de Materiales de Alicante, Universidad de Alicante Alicante 03080 Spain; Organic Optoelectronic Unit, Okinawa Institute of Science and Technology Graduate University 1919-1 Tancha, Onna-son, Kunigami-gun Okinawa 904-0495 Japan; Departamento de Óptica, Farmacología y Anatomía and Instituto Universitario de Materiales de Alicante, Universidad de Alicante Alicante 03080 Spain

## Abstract

We report the synthesis of a dibenzodinaphthocoronene (DBDNC) derivative as a novel nanographene with armchair, zigzag, and fjord edges, which was characterized by NMR and X-ray crystallography as well as infrared (IR) and Raman spectroscopies. Ultrafast transient absorption (TA) spectroscopy revealed the presence of stimulated emission signals at 655 nm and 710 nm with a relatively long lifetime, which resulted in dual amplified spontaneous emission (ASE) bands under ns-pulsed excitation, indicating the promise of DBNDC as a near-infrared (NIR) fluorophore for photonics. Our results provide new insight into the design of nanographene with intriguing optical properties by incorporating fjord edges.

## Abstract 2

Dibenzo[*a*,*m*]dinaphtho[*ef*,*hi*]coronene with zigzag and fjord edges was synthesized and characterized, demonstrating a nonplanar structure with near-infrared stimulated emission with a relatively long lifetime and dual-amplified spontaneous emission.

## Graphical abstract

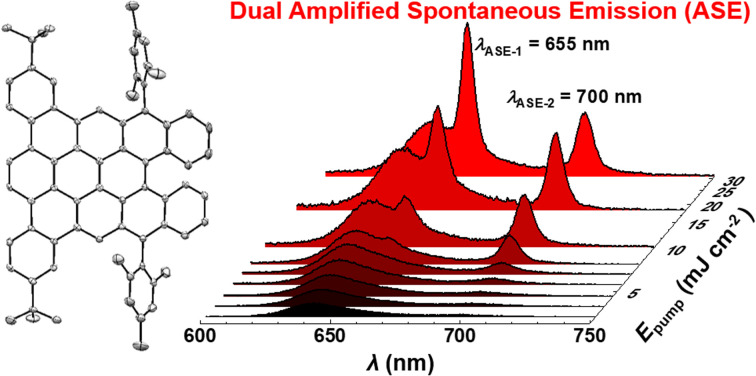

## Introduction

Nanographenes (NGs), namely large polycyclic aromatic hydrocarbons (PAHs), have attracted increasing attention due to their unique optical and electronic properties and high thermal and photostability, which give them the potential for optoelectronic applications.^1–7^ The optical and electronic properties of NGs are dependent on their size, symmetry, and edge structures, which include armchair,^8^ zigzag,^9–17^ cove,^18–20^ and fjord edge structures.^21–24^ While fully armchair-edged NGs have fully benzenoid structures and relatively large energy gaps, as represented by hexa-*peri*-hexabenzocoronene (HBC),^8^ NGs with a combination of zigzag and armchair edges have revealed smaller energy gaps and intriguing optical and magnetic properties.^11^ For example, dibenzo[*hi*,*st*]ovalene (DBOV) was first demonstrated to exhibit amplified spontaneous emission (ASE) and remarkable environmental and operational stability.^25,26^ More recently, fully zigzag-edged NGs with parallelogram shapes have emerged, which also demonstrated ASE and allowed for the fabrication of distributed feedback laser (DFB) devices.^27,28^ However, the spectral overlap between intermolecular charge-transfer excitons and stimulated emission,^3,25,26,29,30^ which is promoted by the effective supramolecular packing of such planar NGs, can hamper the occurrence of long-lived stimulated emission signals. In this regard, the synthesis of nonplanar NGs with stable optical gain has been awaited for photonic applications.

**Fig. 1:**
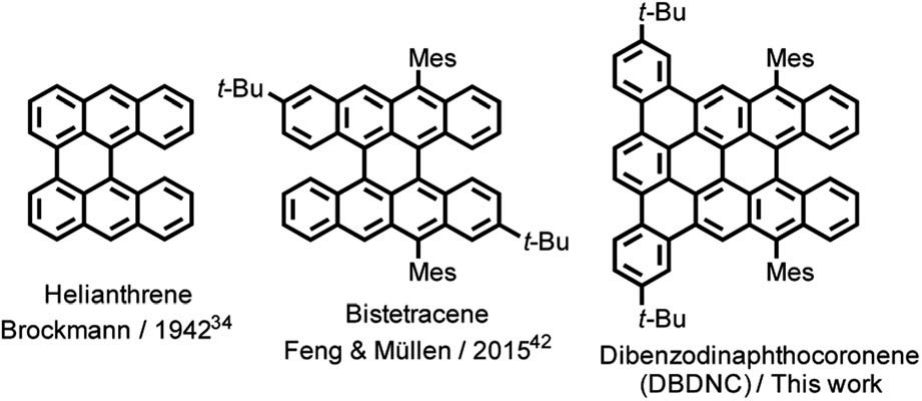
Structures of helianthrene, bistetracene, and DBDNC 1.

In contrast to the planar armchair and zigzag edges, cove and fjord edges, corresponding to [4] and [5] helicenes, respectively, enforce twisted configurations, providing direct access to nonplanar NGs.^5,31^ While a number of cove- or fjord-edged NGs with unique contorted and/or twisted conformations and intriguing (opto)electronic and supramolecular properties have been reported,^31–33^ their combination with extended zigzag edges (three or more benzene rings) has remained rare. Helianthrene, originally reported by H. Brockmann in 1942 (Fig. 1),^34^ can be considered a classical example of this category, which was known for its high reactivity with oxygen to form the endoperoxide.^35–41^ Bistetracene with a combination of zigzag and fjord edges was more recently synthesized to show an open-shell character.^42^ Although the initially reported bistetracene derivative was unstable and oxidized under ambient conditions, kinetic protection with four substituents on the zigzag edges allowed for the stabilization and integration into field-effect transistor devices, demonstrating promising bipolar charge transport properties.^43^

Herein, we report the synthesis and characterization of dibenzo[*a*,*m*]dinaphtho [3,2,1-*ef*:1′,2′,3′-*hi*]coronene (DBDNC) 1 as a novel NG with fjord and extended zigzag edges, which demonstrated high stability and strong red emission. The nonplanar structure of DBDNC 1 was unambiguously revealed by X-ray crystallography, and its vibrational properties were investigated by infrared (IR) and Raman spectroscopies supported by density functional theory (DFT) calculations. Moreover, the in-depth investigations of the photophysical properties of 1 by ultrafast transient absorption (TA) spectroscopy and photoluminescence spectroscopy under ns-pulsed excitation revealed the occurrence of long-lived near-infrared (NIR) stimulated emission transitions as well as dual amplified spontaneous emission.

## Results and discussion

**Fig. 2:**
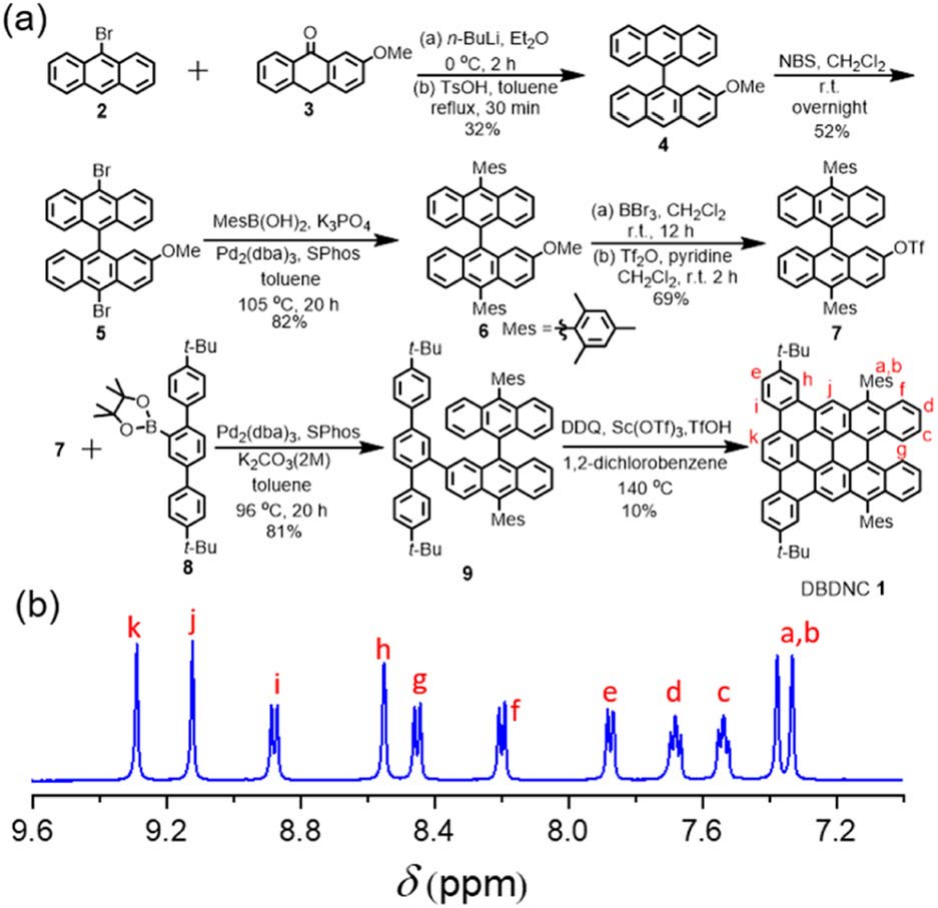
(a) Synthetic route to DBDNC 1; (b) ^1^H NMR spectra (aromatic region) of DBDNC 1 in THF-*d*_8_ : CS_2_ (1 : 1) (500 MHz, 298 K).

For the synthesis of DBDNC 1, 9-bromoanthracene (2) was initially lithiated and reacted with 2-methoxyanthracen-9(10*H*)-one (3)^44^ (Fig. 2). Then, dehydroxylation in the presence of a catalytic amount of *p*-toluenesulfonic acid (TsOH) afforded 2-methoxy-9,9′-bianthracene (4) in 32% yield. Subsequently, bromination of 3 by *N*-bromosuccinimide (NBS) provided 10,10′-dibromo-2-methoxy-9,9′-bianthracene (5) in 52% yield. 10,10′-Dimesityl-2-methoxy-9,9′-bianthracene (6) was obtained by Suzuki–Miyaura coupling of 5 and mesitylboronic acid in 82% yield. Then, 6 was subjected to demethylation by boron tribromide (BBr_3_) and reacted with trifluoromethanesulfonic anhydride (Tf_2_O) to form triflate 7 in 69% yield. Suzuki–Miyaura coupling of 7 with terphenyl boronic ester 8 (see (ESI†) for the preparation of 8) gave precursor 9. Finally, oxidative cyclodehydrogenation of 9 using 2,3-dichloro-5,6-dicyano-1,4-benzoquinone (DDQ), scandium(iii) triflate, and trifluoromethanesulfonic acid (TfOH) in 1,2-dichlorobenzene at 140 °C for 2 h afforded DBDNC 1 in 10% yield.

When the reaction time was extended to 4 h, a signal at *m*/*z* = 920.6 was observed, suggesting the formation of one additional C–C bond, presumably at the fjord region, leading to 10,17-di-*tert*-butyl-7,20-dimesityltetrabenzo [*a*,*g*,*mn*,*pq*]ovalene (TBOV) (Fig. S21†). However, this product could not be isolated due to its low stability, being prone to oxidation (Fig. S22†), precluding the structural characterization. Well-resolved ^1^H and ^13^C NMR spectra of DBDNC 1 could be recorded in a mixture of tetrahydrofuran (THF) and carbon disulfide (CS_2_), and the aromatic proton signals could be assigned with ^1^H–^1^H correlation spectroscopy (COSY) measurements (Fig. 2 and S17–S19†).

**Fig. 3:**
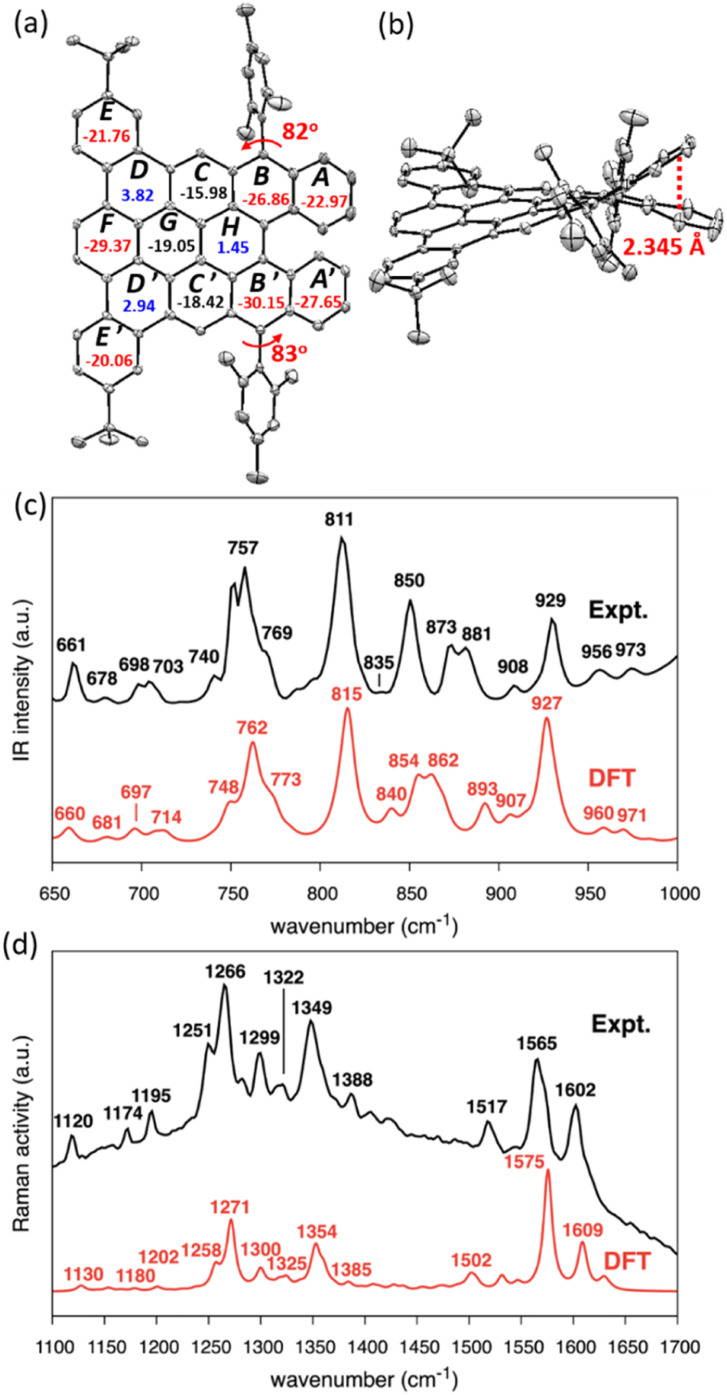
X-ray crystal structure of DBDNC 1: (a) top and (b) side views. Hydrogen atoms and solvent molecules are omitted for clarity. Ellipsoids are drawn at the 50% probability level. The numbers inside the rings in panel (a) indicate NICS(1)_*zz*_ values. (c and d) Measured (black line) and calculated (red line) (c) IR and (d) Raman spectra of DBDNC 1. Selected peaks are labeled with their wavenumbers (cm^−1^). The wavenumbers computed by DFT were uniformly scaled by 0.98.

The single crystal of DBDNC 1 was obtained through evaporation of its solution in benzene/methanol, enabling structural analysis by X-ray crystallography. The structure of DBDNC 1 with the nonplanarity induced by the fjord edge was thus clearly demonstrated. The vertical distance of fjord-edged periphery between the centroids of rings *A* and *A*′ is in the range of 2.345 Å, resulting in a torsion angle of ∼49.5° (Fig. 3a, b and S23c†). The two mesityl groups are tilted with dihedral angles of 82°–83°. In the packing structure of DBDNC 1, there are two pairs of enantiomers with a racemate of (*P*)–1 and (*M*)–1 in the unit cell (Fig. S23b†). No obvious intermolecular π–π interaction was observed apparently due to the existence of nonplanar fjord edges as well as the *tert*-butyl and mesityl groups at the peripheral positions. To investigate the aromaticity of DBDNC 1, nucleus-independent chemical shifts (NICS)^45^ were calculated at GIAO-B3LYP/6-31G(d,p) level of theory. As shown in Fig. 3a, rings *A*, *A*′, *B*, and *B*′ displayed large negative NICS(1)_*zz*_ values (red color), indicating strong aromaticity at the fjord region. The NICS(1)_*zz*_ values of rings *D*, *D*′ and *H* are 3.82, 2.94, and 1.45 (blue color), respectively, which suggests non-aromatic characters. This result agreed with an anisotropies of the induced current density (ACID)^46,47^ plot calculated for non-substituted DBDNC, only including the π-electrons, which showed interruption of the diatropic ring currents at the *D*, *D*′ and *H* rings (Fig. S25†).

The IR and Raman spectra were recorded on a powder sample of DBDNC 1 and compared with the DFT-simulated spectra, displaying good agreement (Fig. 3). The out-of-plane CH bending region of the IR spectrum of DBDNC 1 showed significant vibrational fingerprints that can be associated with the molecular structure through the topology of the CH groups placed at the edge of the aromatic moieties, classified as SOLO, DUO, and QUATRO (Fig. 3c; see the ESI† for details).^48^ Briefly, the strong IR peak at 757 cm^−1^ (762 cm^−1^, scaled, DFT) could be assigned to the QUATRO mode, corresponding to the fjord edge. The DUO and SOLO modes from the armchair and zigzag edges were found at 811 and 873 cm^−1^ (815 and 862 cm^−1^, scaled, DFT), respectively. Moreover, other calculated IR-active normal modes of DBDNC 1 were also carefully inspected to obtain the band assignments summarized in Table S3,† with the graphical representation of the most IR-active modes in Table S4.† The measured and calculated Raman spectra of DBDNC 1 show a D band at approximately 1300 cm^−1^ and a G band near 1600 cm^−1^ (Fig. 3d), which account for ring-breathing and ring-stretching modes, respectively, in the aromatic core of DBDNC 1 (Fig. S27†). The assignment of the representative Raman-active vibrational normal modes of DBDNC 1 are summarized in Tables S5 and S6.†

The electrochemical properties of DBDNC 1 were investigated by cyclic voltammetry (CV) in dichloromethane with ferrocene as an external standard (Fig. S28†). We observed two reversible oxidation peaks and one reversible reduction, with half-wave potentials respectively at *E*_1/2ox_ = +0.05 V and +0.61 V and at *E*_1/2red_ = −1.69 V with reference to Fc/Fc^+^. The HOMO and LUMO energy levels were estimated to be −4.85 eV and −3.17 eV based on the onsets of the oxidation and reduction peaks, respectively. Thus, the electrochemical energy gap was determined to be 1.68 eV.

**Fig. 4:**
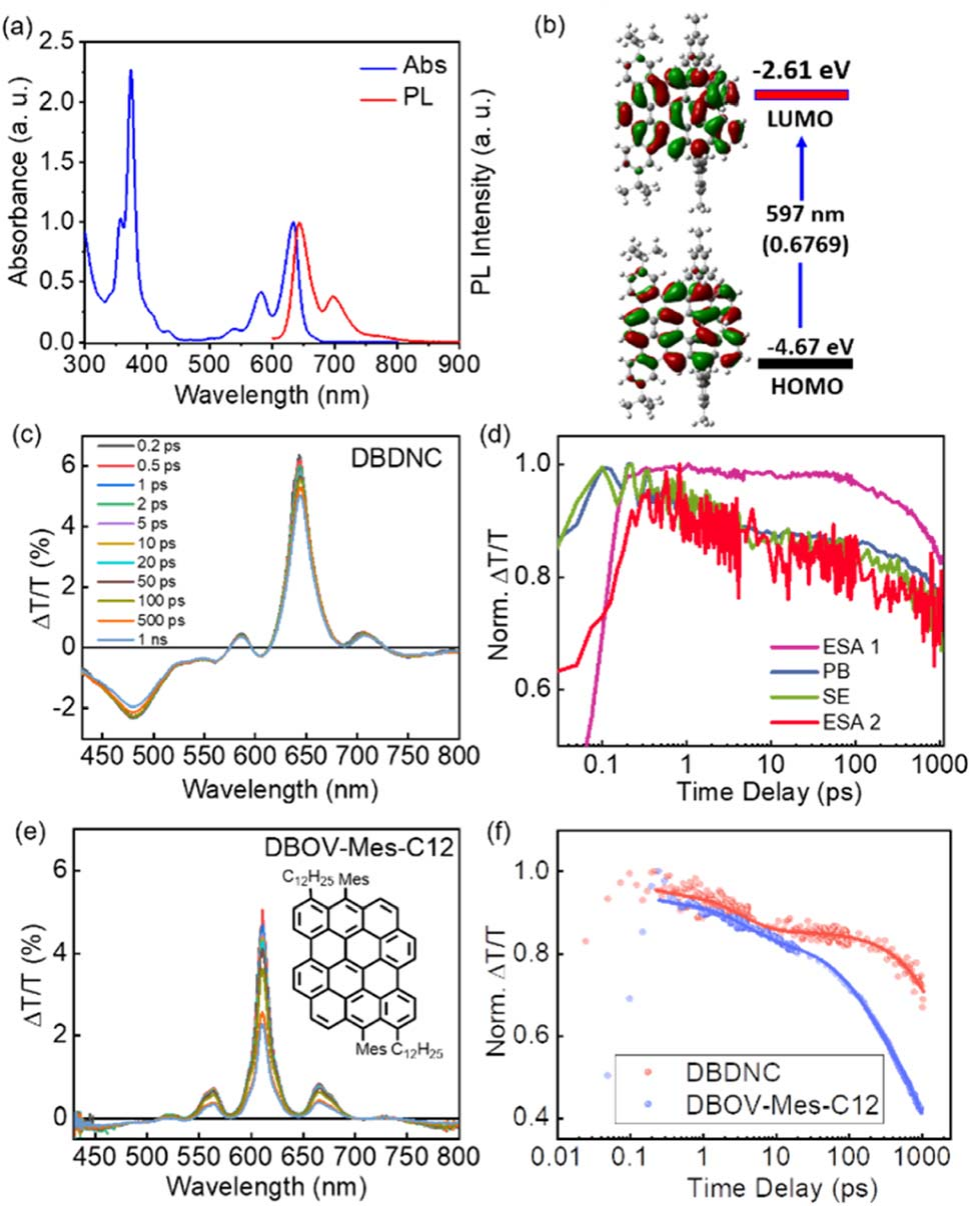
(a) Normalized absorption and fluorescence spectra of DBDNC 1 in toluene (10^−5^ M). (b) Frontier molecular orbitals and energy diagrams of 1 calculated by TD-DFT at the CAM-B3LYP/6-311G(d,p) level. Values in parentheses represent the oscillator strengths (*f*). (c) TA spectra and (d) dynamics (excitation 630 nm) of DBDNC 1 (0.1 mg mL^−1^ in toluene). (e) TA spectra (excitation at 630 nm) of DBOV-Mes-C12 (0.1 mg mL^−1^ in toluene; inset: the structure of DBOV-Mes-C12). (f) Comparison between the dynamical traces of the SE signal in DBDNC 1 (probe at 710 nm, biexponential fitting *τ*_1_ = 3 ps, *τ*_2_ = 6 ns) and DBOV-Mes-C12 (probe at 660 nm, triexponential fitting *τ*_1_ = 3 ps, *τ*_2_ = 170 ps, *τ*_3_ = 2.2 ns). The dots represent the experimental data, while the solid lines represent the fitting modes. The TA data of DBOV-Mes-C12 are taken from our previous experiments.^25^ The *x*-scale in the dynamical traces is reported in logarithmic units.

The UV/vis absorption spectrum of DBDNC 1 in toluene was clearly structured with maxima at 633, 582, and 373 nm (Fig. 4a), which agreed with simulation by TD-DFT calculations at the CAM-B3LYP/6-311G(d,p) level (Fig. S24†). The long-wavelength absorption maximum at 633 nm was assigned to the HOMO → LUMO transition calculated at 597 nm (*f* = 0.6769) (Fig. 4b and Table S1†). Notably, the solution of DBDNC 1 in toluene showed negligible changes in the UV/vis spectrum over half a month at room temperature in ambient conditions (Fig. S29†), indicating its high stability. The emission spectrum of DBDNC 1 in toluene displayed well-defined peaks at 643, 698, and 772 nm, which were assigned to the 0–0, 0–1, and 0–2 vibronic bands, respectively. The optical energy gap was estimated from the interface of the absorption and emission spectra to be ∼1.94 eV. The Stokes shift was as small as 10 nm (246 cm^−1^), which is a clear signature of the rigid structure of this nanographene molecule notwithstanding the fjord edge. The absolute photoluminescence quantum yield (PLQY; *Φ*) was 53%, and the fluorescence lifetime (*τ*_s_) was determined to be 8.5 ns (Fig. S30†). According to the equations *Φ* = *k*_r_ × *τ*_s_ and *k*_r_ + *k*_nr_ = *τ*_s_^−1^, the radiative (*k*_r_) and nonradiative (*k*_nr_) decay rate constants from the singlet excited state were determined to be 6.2 × 10^7^ s^−1^ and 5.5 × 10^7^ s^−1^, respectively.

We employed TA spectroscopy to gain further insights into the photophysics of DBDNC 1. Specifically, we observe a transient spectrum that includes an excited state absorption (ESA) in the visible range (ESA 1, 430–560 nm), a photobleaching (PB) signal convoluted with stimulated emission (SE, 0′ → 0) at 640 nm, a positive peak corresponding to SE (0′ → 1) at 710 nm, and another ESA in the NIR range at 730–800 nm (ESA 2) (Fig. 4c and d shows the spectra and the associated time-decays, respectively). Although the spectral features resemble those of NGs with zigzag edges, including DBOV derivatives,^3,26,29^ DBDNC 1 exhibits distinct features, especially in terms of SE. First, this signal is appreciably redshifted toward the NIR region (*i.e.*, 660 nm *vs.* 710 nm in DBOV-Mes-C12 (ref. ^25^) and DBDNC 1, respectively, Fig. 4e). This effect can be attributed to the relatively small optical gap (1.94 eV) of DBDNC 1. Furthermore, we note that the SE lifetime is appreciably longer in DBDNC 1 than in DBOV-Mes-C12 (Fig. 4f), although the signal of the former would be affected more by the energy gap law than the latter, owing to the longer SE wavelength.^49^ In particular, for DBDNC 1 the SE relaxation can be modelled *via* two-exponentials (3 ps and 6 ns, due to internal conversion and radiative relaxation from the first excited singlet S_1_), while in DBOV-Mes-C12 we have an additional fitting component (170 ps) that can be attributed to the absorption losses due to charge absorption in aggregates.^30^ In addition, the relaxation from S_1_ is sensibly longer in DBDNC 1 than in DBOV-Mes-C12 (*i.e.*, 6 ns *vs.* 2 ns) likely owing to the enhanced electron delocalization in the former, which usually leads to an increase of the emission lifetime in NGs.^50^

**Fig. 5:**
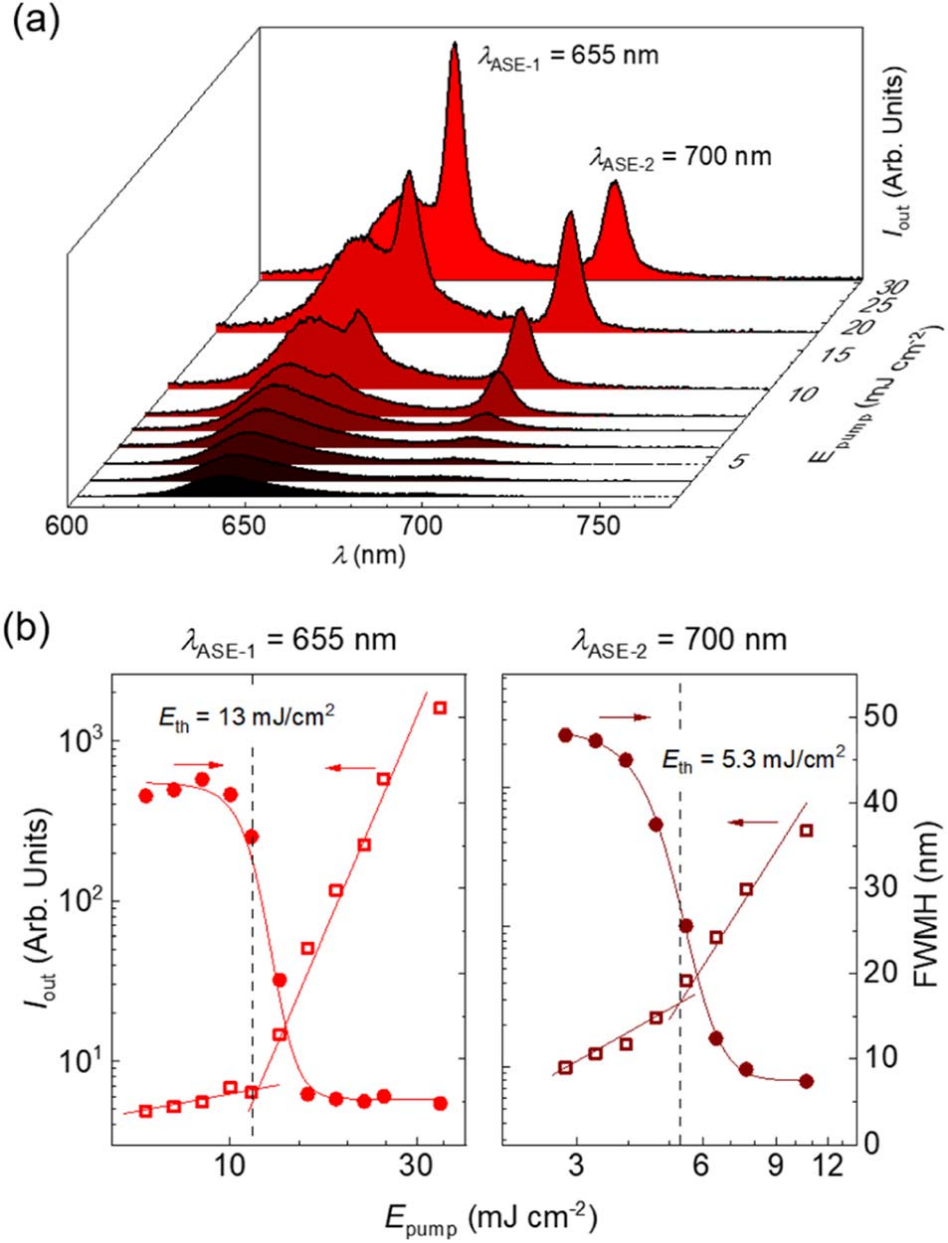
(a) Emission spectra of DBDNC 1 dispersed at 1 wt% in a polystyrene thin film captured at different pump energy densities (*E*_pump_). ASE peaks appear at *λ*_ASE-1_ = 655 nm and *λ*_ASE-2_ = 700 nm. (b) Representations of the output intensity (*I*_out_; open squares, left axis) and emission linewidth, defined as the full width at half maximum, (FWHM; full circles, right axis) *versus E*_pump_ used to determine the ASE thresholds (*E*_th_). Vertical dashed lines are guides to the eye.

Remarkably, polystyrene (PS) thin films doped with 1 wt% of DBDNC 1 demonstrated dual amplified spontaneous emission (ASE) under ns-pulsed excitation at 355 nm (Fig. 5a). The presence of ASE is a clear signature of optical amplification, which can be identified by the collapse of the broad PL spectrum into a narrow peak accompanied by the sudden increase of the output intensity (*I*_out_) upon increasing the pump fluence. Two different narrow ASE bands are observed at 655 and 700 nm with different threshold (*E*_th_) values of 13 and 5.3 mJ cm^−2^, respectively (Fig. 5b). These threshold values are high compared with state-of-the-art NGs used for lasing (30–300 μJ cm^−2^),^26,28,51^ which present a single ASE band matching their second vibronic. Nevertheless, dual-ASE in NGs has rarely been reported, with FZ3 (ref. 50) and PP-Ar^52^ being the only two known reported examples, to the best of our knowledge.

The ASE performance of 1 is comparable to that of these two NGs (see Table S8†) and might be attributed to the existence of two vibronic progressions associated to different vibrational modes. The first ASE peak might correspond to a low frequency bending mode (∼240 cm^−1^) that overlaps with the first vibronic. Meanwhile, the second ASE peak might correspond to a collective aromatic CC stretching mode (∼1290 cm^−1^). These frequencies have been estimated from the energy difference between the first PL vibronic (643 nm) and each ASE peak (655 and 700 nm). Additionally, the ESA band overlapped with SE at 710 nm plays an important role. Typically, SE can be found at both vibronic progressions, but reabsorption is much higher for the peak at 655 nm that is closer to the ground-state absorption band. Then, ASE is observed only from the peak associated to the CC stretching mode (700 nm) because there is a large difference between both thresholds. However, in the case of DBDNC 1, the detrimental effect of the ESA band introduces extra losses that compensate the threshold difference and both ASE bands become active. Besides, ground-state absorption remains still higher than ESA, which accounts for the threshold difference between both ASE bands. These detrimental effects associated to reabsorption and ESA certainly account for the higher thresholds of DBDNC 1 compared with state-of-the-art NGs, in which ASE is observed only from the second vibronic associated to the aromatic CC stretching mode with only a little or negligible presence of reabsorption and/or ESA.

Finally, the ASE photostability under ns-pulsed excitation was studied by pumping the films uninterruptedly at the same position with a pump fluence twice the ASE threshold (Table S8 and Fig. S32†). The photostability half-life parameter is used to quantify the performance, which is defined as the time or number of pulses needed for the output intensity to become half its initial value. The PS films doped with DBDNC 1 show lower photostability (*t*_1/2_ = 400 pump pulses [40 s]) than other NGs with similar ASE performance (*t*_1/2_ ∼ 10^5^ pump pulses for FZ3 and PP-Ar).^49,51^ Such difference might be due to a higher reactivity toward oxygen, considering the helianthrene substructure, or higher intersystem crossing rate associated to the fjord edge.

## Conclusions

In summary, we have achieved the synthesis of DBDNC 1 as a novel NG with fjord and extended zigzag edges and a rare π-extended analog of helianthrene. The nonplanar structure of DBDNC 1 was unambiguously elucidated by X-ray crystallography, and further confirmed by IR and Raman spectroscopies supported by DFT calculations, which also revealed the intriguing vibrational fingerprints precisely assignable, *e.g.*, to fjord, zigzag, and armchair edges. DBDNC 1 displayed a narrow optical energy gap (∼1.94 eV) and strong emission with the PLQY of 54%. Moreover, DBDNC 1 demonstrated NIR SE with a relatively long lifetime, which was not affected by the intermolecular charge transfer, in contrast to the planar DBOV-Mes-C12. Remarkably, PS thin films doped with DBDNC 1 exhibited dual ASE at 655 and 700 nm under ns-pulsed excitation, showing its potential for applications in NIR photonics. This work also offers new insight for the design of a further variety of non-planar NGs with zigzag and fjord edges with intriguing photophysical properties.

## Data availability

Crystallographic data for DBDNC 1 has been deposited at the Cambridge Crystallographic Data Centre (CCDC number: 2167126) and can be obtained from www.ccdc.cam.ac.uk/structures. Other data that support the findings of this study are available within the manuscript and in the ESI† or from the corresponding authors upon reasonable request.

## Author contributions

X. X. synthesized all the compounds and performed standard characterization under the supervision by A. N. G. S., A. L. and M. T. carried out the IR and Raman studies. A. V., F. S. and G. M. P. performed the transient absorption studies. R. M.-M., M. G., P. G. B. and M. A. D.-G. conducted the ASE studies. S. V. carried out the single-crystal X-ray diffraction analysis. Z. L. and R. K. performed the time-resolved photoluminescence analysis. X. X. and A. N. wrote the manuscript with contributions by G. S., R. M.-M., M. T., M. A. D.-G., F. S. and G. M. P, and finalized it with comments by other co-authors.

## Conflicts of interest

There are no conflicts to declare.

## Supplementary Material

SC-013-D2SC04208H-s001

SC-013-D2SC04208H-s002
