# Supplementary material for: Synthesis of zigzag- and fjord-edged nanographene with dual amplified spontaneous emission
Source: Chem Sci. 2022 Oct 18;13(44):13040–5. doi: 10.1039/d2sc04208h (PMC9667923; doi:10.1039/d2sc04208h)
Supplement: SC-013-D2SC04208H-s001 [file SC-013-D2SC04208H-s001.pdf]

Electronic Supplementary Material (ESI) for Chemical Science.
This journal is © The Royal Society of Chemistry 2022

Supporting Information for Synthesis of Zigzag- and Fjord-Edged Nanographene with Dual Amplified Spontaneous Emission

Xiushang Xu,[a,b] Gianluca Serra,[c] Andrea Villa,[d] Rafael Muñoz-Mármol,[d] Serhii Vasylevskyi,[e] Marcos Gadea,[f] Andrea Lucotti,[c] Zensen Lin,[g] Pedro G. Boj,[h] Ryota Kabe,[g] Matteo Tommasini,[c] María Á. Díaz-García,[f] Francesco Scotognella,[d] Giuseppe Maria Paternò,*[,d] Akimitsu Narita*[,a,b]

[a] Organic and Carbon Nanomaterials Unit, Okinawa Institute of Science and Technology Graduate University, 1919-1 Tancha, Onna-son, Kunigami-gun, Okinawa 904-0495, Japan, E-mail: akimitsu.narita@oist.jp

[b] Max Planck Institute for Polymer Research, Ackermannweg 10, 55128 Mainz, Germany

[c] Dipartimento di Chimica, Materiali e Ingegneria Chimica 'G. Natta', Politecnico di Milano, Piazza Leonardo da Vinci 32, 20133 Milano, Italy

[d] Physics Department, Politecnico di Milano, Piazza L. da Vinci 32, Milano 20133, Italy, E-mail: giuseppemaria.paterno@polimi.it

[e] Engineering Section, Research Support Division, Okinawa Institute of Science and Technology Graduate University, 1919-1 Tancha, Onna-son, Kunigami-gun, Okinawa 904-0495, Japan

[f] Departamento de Física Aplicada and Instituto Universitario de Materiales de Alicante, Universidad de Alicante, Alicante 03080, Spain

[g] Organic Optoelectronic Unit, Okinawa Institute of Science and Technology Graduate University, 1919-1 Tancha, Onna-son, Kunigami-gun, Okinawa 904-0495, Japan

[h] Departamento de Óptica, Farmacología y Anatomía and Instituto Universitario de Materiales de Alicante, Universidad de Alicante, Alicante 03080, Spain

1. General experimental details

All reactions working with air- or moisture-sensitive compounds were carried out under argon atmosphere using standard Schlenk line techniques. Unless otherwise noted, all starting materials and other chemicals were purchased from commercial sources and used without further purification. 2-Methoxyanthracen-9(10H)-one was prepared following our previous procedure[1]. Thin-layer chromatography (TLC) was done on silica gel coated aluminum sheets with F254 indicator and column chromatography separation was performed with silica gel (particle size 0.063–0.200 mm). Analytical and preparative high-performance liquid chromatography (HPLC) was performed on Shimadzu HPLC systems equipped with COSMOSIL Buckyprep Packed Columns (Nacalai Tesque Inc.). Nuclear Magnetic Resonance (NMR) spectra were recorded using Bruker DPX, 300MHz, 400 MHz, 500 MHz NMR spectrometers. Chemical shifts (*δ*) were expressed in ppm relative to the residual of solvents (CD_2Cl_2, ^{1}H: 5.32 ppm, ^{13}C: 53.84 ppm; $CDCl_3$, ^{1}H: 7.26 ppm, ^{13}C: 76.99 ppm, THF-d_8, ^{1}H: 1.72 ppm, 3.57 ppm, ^{13}C: 24.40 ppm, 66.43 ppm). Coupling constants (*J*) were recorded in Hertz. High-resolution mass spectra (HRMS) were recorded on a Bruker Reflex II-TOF spectrometer by matrix-assisted laser decomposition/ionization (MALDI), using 7,7,8,8-tetracyanoquinodimethane (TCNQ) as matrix and by calibrating with poly(ethylene glycol), or on a RED-00000556 Mass Spectrometer Electrospray ionization (ESI) Thermo Orbitrap Thermo Scientific LTQ-Orbitrap. Other MALDI-TOF MS analyses were performed on Shimadzu MALDI-8020 mass spectrometer using TCNQ as the matrix.

The FT-IR spectrum of compound DBDNC **1** was measured with a Nicolet Nexus equipment coupled with a Thermo-Nicolet Continuum infrared microscope and a cooled MCT detector (77 K). The micro-FT-IR spectrum was collected in transmission mode by depositing the powder sample obtained from the synthesis on a diamond anvil cell (4 cm^{-1} spectral resolution). The reported FT-Raman spectrum of DBDNC **1** was acquired with the Nicolet NXR 9650 instrument equipped with a Nd-YVO4 laser excitation (1064 nm) and an InGaAs detector. The powder sample of DBDNC **1** was deposited on a metallic support and analysed with the micro stage FT-Raman setup (spot size 50 μm, 2W laser power, 4 cm^{-1} spectral resolution).

For cyclic voltammetry (CV) measurements, tetrabutylammonium hexafluorophosphate (n-Bu_4NPF_6) from TCI was used as a supporting electrolyte after recrystallization from ethanol and then dissolved in dichloromethane (DCM) from Sigma-Aldrich (HPLC grade, used as received). Electrochemical experiments were carried out on a BAS ALS610E in a three-electrode cell in the dichloromethane solution of n-Bu_4NPF_6 (0.1 M) at a scan rate of 50 mV/s at room temperature. A Pt wire, $Ag/AgNO_3$, and a glassy carbon electrode were used as the counter electrode, the reference electrode, and the working electrode, respectively. All the $E_{1/2}$ potentials were directly obtained from cyclic voltammetric curves as averages of the cathodic and anodic peak potentials and determined by the ferrocenium/ferrocene couple (Fc^+/ Fc) standard potential. The HOMO and LUMO energy levels were calculated according to the following equations: HOMO = $-(4.8+E_{ox}^{onset})$; LUMO = $-(4.8+E_{red}^{onset})$, where E_{ox}^{onset} and E_{red}^{onset} onset referred to the onset potentials of the first oxidative and reductive redox waves, respectively.

Photoluminescence lifetime was recorded using a streak camera system (C14832-110, Hamamatsu Photonics) equipped with a 300mm triple grating imaging spectrograph (SpectraPro, HRS-300-SS, Princeton Instruments) and excitation was provided by a Yb:KGW femtosecond laser (PHAROS, Light Conversion). Photoluminescence quantum yields (PLQY) were measured using an integrating sphere with a photoluminescence measurement unit (Quantaurus-QY, C11347-01, Hamamatsu Photonics).

2. Synthetic details

2-methoxy-9,9'-bianthracene (**4**)

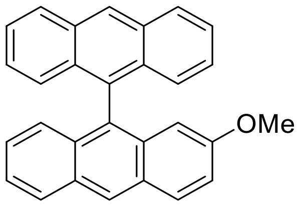

To a solution of 9-bromoanthracene (2.74 g, 10.7 mmol) in anhydrous diethyl ether (100 mL) was added dropwise *n*-butyllithium (*n*-BuLi, 1.6 M in hexane, 7.0 mL, 11 mmol) under argon atmosphere at 0 ˚C. After stirring for 10 min, 2-methoxyanthracen-9(10*H*)-one (**3**) was added, and the reaction mixture was stirred for another 2 h at room temperature. The reaction was then quenched with methanol (10.0 mL), and concentrated in vacuo. The residue was dissolved in toluene (30 mL), and refluxed with a catalytic amount of *p*-toluenesulfonic acid for 30 min. After cooling to room temperature, the reaction mixture was washed with brine and extracted with CH_2Cl_2 (30 mL) for three times. The organic layers were combined, dried with anhydrous $MgSO_4$, and concentrated. The crude product was purified by silica gel column chromatography (eluent: hexane: CH_2Cl_2 = 5:1) to afford the title compound (1.12 g, 32% yield) as a pale white solid. ^{1}H NMR (400 MHz, $CDCl_3$) δ 8.72 (s, 1H) 8.64 (s, 1H), 8.21 – 8.12 (m, 2H), 8.10 (d, J = 8.5 Hz, 1H), 8.05 (d, J = 9.2 Hz, 1H), 7.45 (m, 2H), 7.38 (m, 1H), 7.18 – 7.09 (m, 5H), 6.97 (m, 1H), 6.23 (d, J = 2.5 Hz, 1H), 3.29 (s, 3H). ^{13}C NMR (75 MHz, CD_2Cl_2) δ 157.99, 133.59, 133.02, 132.39, 132.11, 131.76, 131.01, 130.73, 130.65, 129.05, 128.58, 127.61, 126.92, 126.44, 126.33, 126.17, 125.69, 124.76, 103.03, 55.18. HRMS (ESI, Positive): *m*/*z* Calcd. For $C_{29}H_{21}O$: 385.1587 $[M+H]^+$, found: 385.1570.

10,10’-dibromo-2-methoxy-9,9’-bianthracene (**5**)

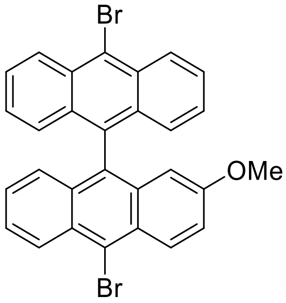

A 250-mL round-bottom flask was charged with compound **4** (15.0 mg, 0.0390 mmol), *N*-bromosucinimide (NBS) (14.5 mg, 0.0818 mmol), and CH_2Cl_2 (10 mL). After stirring for overnight at room temperature, the reaction was quenching with acetone (5 mL), and the solvents were evaporated. The residue was purified by silica gel column chromatography (eluent: hexane: CH_2Cl_2 = 10:1) to give the title compound (10.0 mg, 48% yield) as the yellow solid. ^{1}H NMR (400 MHz, $CDCl_3$) δ 8.78 – 8.66 (m, 2H), 8.67 – 8.57 (m, 2H), 7.58 (ddt, J = 8.9, 6.4, 1.4 Hz, 2H), 7.54 – 7.45 (m, 1H), 7.28 (dd, J = 2.7, 1.3 Hz, 1H), 7.23 – 7.08 (m, 5H), 6.94 (d, J = 8.4 Hz, 1H), 6.22 (t, J = 1.9 Hz, 1H), 3.30 (d, J = 1.3 Hz, 3H). ^{13}C NMR (101 MHz, $CDCl_3$) δ 157.76, 133.60, 133.32, 132.70, 132.04, 130.72, 130.55, 130.05, 129.13, 128.17, 128.12, 127.27, 127.14, 126.64, 126.47, 126.33, 126.23, 124.01, 123.83, 121.96, 103.01, 55.13. HR MS (ESI, Positive): *m*/*z* Calcd. For $C_{29}H_{18}Br_2O$: 540.9797 $[M+H]^+$, found: 539.9768.

10,10'-dimesityl-2-methoxy-9,9'-bianthracene (**6**)

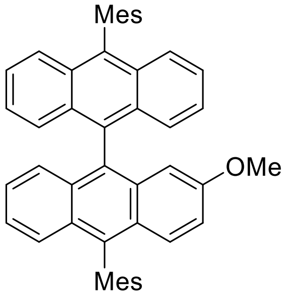

A 100-mL Schlenk tube was charged with compound **5** (20.0 mg, 0.0369 mmol), 2,4,6-trimethylphenylboronic acid (18.2 mg, 0.111 mmol), tris(dibenzylideneacetone)dipalladium(0) ($Pd_2(dba)_3$) (3.30 mg, 3.69 μmol), 2-dicyclohexylphosphino-2',6'-

dimethoxybiphenyl (SPhos) (3.02 mg, 7.38 µmol), K_3PO_4 (47.2 mg, 0.222 mmol), and anhydrous toluene (4 mL) under argon atmosphere. The reaction mixture was subjected to freeze-pump-thaw cycles (three times) and heated at 120 ºC for 20 h under argon atmosphere. After the resulting mixture was cooled to room temperature and poured into water (50 mL), the organic layer was separated, and the aqueous layer was extracted with CH_2Cl_2 (20 mL) for three times. The separated organic phases were combined, washed with brine, dried over anhydrous $MgSO_4$, and evaporated. The residue was purified by silica gel column chromatography (eluent: hexane: CH_2Cl_2 = 10:1) to give the title compound (17.2 mg, 75% yield) as light yellow solid. ^{1}H NMR (500 MHz, CD_2Cl_2) δ 7.62 – 7.47 (m, 3H), 7.44 (d, J = 9.4 Hz, 1H), 7.25 (t, J = 6.3 Hz, 2H), 7.20 (m, 1H), 7.18 – 7.04 (m, 10H), 6.91 (dt, J = 9.4, 1.6 Hz, 1H), 6.13 (d, J = 2.6 Hz, 1H), 3.13 (s, 3H), 2.41 (m, 6H), 1.90 (s, 3H), 1.83 (s, 6H), 1.71 (s, 3H). ^{13}C NMR (126 MHz, CD_2Cl_2) δ 157.25, 137.49, 137.39, 136.71, 136.47, 134.74, 134.66, 133.18, 132.68, 132.05, 131.37, 130.62, 129.78, 128.28, 128.18, 127.00, 126.69, 126.45, 126.28, 125.78, 125.62, 124.61, 120.05, 103.00, 54.36, 31.60, 22.67, 21.01, 19.88, 19.46, 13.90. HRMS (ESI, Positive): 621.3152 [M+H]$^+$, found: 621.3162.

10,10'-dimesityl-2-trifluoromethanesulfonyloxy-9,9'-bianthracene (**7**)

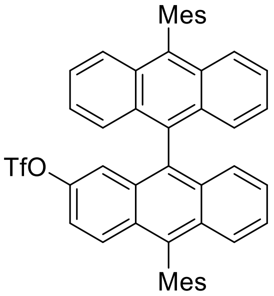

To a solution of compound **6** (500 mg, 806 µmol) in 50 mL of CH_2Cl_2 was added boron tribromide (BBr_3) (1.0 M in CH_2Cl_2, 8.1 mL, 8.1 mmol) dropwise at 0 °C under the argon atmosphere. The resulting mixture was allowed to warm to room temperature and stirred for 12 h. The reaction mixture was poured into water (100 mL), and then extracted with CH_2Cl_2 (20 mL) for three times. The organic layers were combined, washed with brine, dried over $MgSO_4$, and evaporated. The crude compound was dissolved in anhydrous CH_2Cl_2 (20 mL) and cooled to 0 °C. Then, pyridine (0.131 mL, 1.61 mmol) was added into the solution dropwise at 0 °C. After stirring for 10 min, trifluoromethanesulfonic anhydride (Tf_2O) (1.0 M in CH_2Cl_2, 1.2 mL, 1.2 mmol) was added at 0 °C under an argon atmosphere. The resulting mixture was allowed to warm to room temperature and stirred for 2 h. The resulting solution was poured into 50 mL of water and was extracted with CH_2Cl_2 (20 mL) for three times. The organic layers were combined, washed with brine, dried over $MgSO_4$, and evaporated. The residue was purified by silica gel column chromatography to afford the title compound (410 mg, 69% yield) as the light yellow solid. ^{1}H NMR (300 MHz, CD_2Cl_2) δ 7.83 - 7.63 (m, 4H), 7.48 - 7.31 (m, 5H), 7.28 - 7.14 (m, 9H), 6.97 (d, J = 2.6 Hz, 1H), 2.54 (s, 6H), 2.00 (s, 3H), 1.95 (s, 6H), 1.87 (s, 3H). ^{13}C NMR (75 MHz, CD_2Cl_2) δ 147.70, 138.30, 138.01, 137.95, 137.90, 137.86, 137.75, 134.91, 134.51, 134.17, 132.87, 131.87, 131.55, 131.42, 130.87, 130.18, 130.09, 128.89, 128.82, 128.75, 128.68, 127.59, 127.16, 126.99, 126.93, 126.81, 126.38, 126.01, 120.14, 118.32, 30.08, 23.08, 21.39, 20.29, 19.82, 14.26. HRMS (ESI, Positive)*m/z* Calcd. For $C_{47}H_{41}F_3NO_3S$: 756.2754 [M+NH$_4$]$^+$, found: 756.2786.

4,4''-di-*tert*-butyl-2'-(4',4',5',5'-tetramethyl-1',3',2'-dioxaborolan-2'-yl)-1,1':4',1''-terphenyl (**8**)

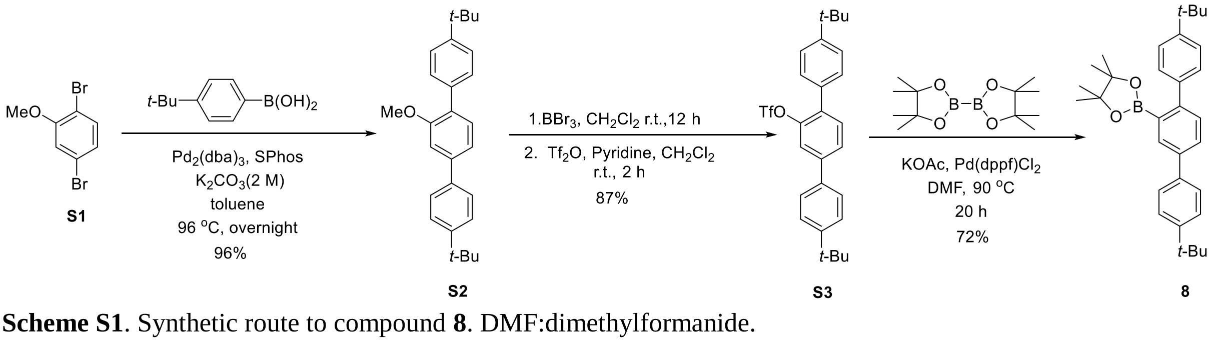

Scheme S1. Synthetic route to compound **8**. DMF:dimethylformanide.

For the synthesis of compound **8**, 4,4''-di-*tert*-butyl-2'-methoxy-1,1':4',1''-terphenyl (**S2**) was prepared by Suzuki-Miyaura coupling of 1,4-dibromo-2-methoxybenzene (**S1**) and 4-*tert*-butylphenylboronic acid in 96% yield. Then, **S2** was subjected to demethylation by BBr_3 in CH_2Cl_2 at 0 °C. The resulting terphenyl was reacted with trifluoromethanesulfonic anhydride (Tf_2O) to form 4,4''-di-*tert*-butyl-2'-trifluoromethanesulfonyloxy-1,1':4',1''-terphenyl (**S3**). Finally, **S3** was converted to **8** by Miyaura-Ishiyama borylation reaction in 72% yield.

4,4''-di-*tert*-butyl-2'-methoxy-1,1':4',1''-terphenyl (**S2**)

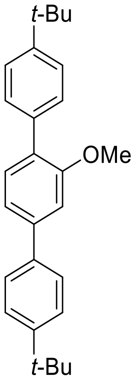

A 250-mL flask was charged with 1,4-dibromo-2-methoxybenzene (6.50 g, 24.6 mmol) and 4-*tert*-butylphenyl boronic acid (9.65 g, 54.2 mmol), $Pd_2(dba)_3$ (1.10 g, 1.23 mmol), SPhos (984 mg, 2.40 mmol), K_2CO_3 (2 M, 5.0 mL) and toluene (20 mL). After degassing by argon bubbling for 15 min, the reaction mixture was heated to be 96 °C under argon atmosphere overnight. After cooling to room temperature, the resulting mixture was extracted with CH_2Cl_2 (50 mL) for three times. The separated organic phases were combined, washed with brine, dried over $MgSO_4$, and evaporated. The residue was purified by silica gel column chromatography (eluent: hexane: CH_2Cl_2 = 5:1) to give the title compound (9.20 g, 96% yield) as the white solid. ^1H NMR (400 MHz, CD_2Cl_2) δ 7.68 - 7.61 (m, 2H), 7.57 - 7.52 (m, 4H), 7.51 - 7.47 (m, 2H), 7.41 (d, J = 7.8 Hz, 1H), 7.29 (dd, J = 7.8, 1.7 Hz, 1H), 7.25 (d, J = 1.7 Hz, 1H), 3.92 (s, 3H), 1.42 (s, 18H). ^{13}C NMR (101 MHz, CD_2Cl_2) δ 156.86, 150.65, 149.89, 141.41, 138.00, 135.38, 130.91, 129.11, 126.61, 125.76, 124.93, 119.31, 109.92, 55.49, 34.44, 31.08. HRMS (ESI, Positive): *m/z* Calcd. For $[C_{27}H_{33}O]^+$: 373.2526 $[M+H]^+$, found: 373.2517.

4,4''-di-*tert*-butyl-2'-trifluoromethanesulfonyloxy-1,1':4',1''-terphenyl (**S3**)

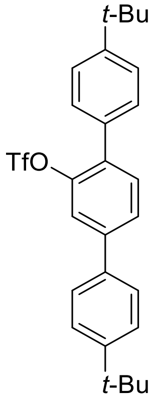

In a 250-mL flask, BBr_3 (1.0 M in CH_2Cl_2, 20.2 ml, 20.2 mmol) was added into the solution of **S1** (2.50 g, 6.72 mmol) in anhydrous CH_2Cl_2(40 mL) at 0 °C under argon atmosphere. The reaction mixture was allowed to warm to room temperature and stirred for 12 h. Then, the reaction mixture was poured into water (100 mL), and then extracted with CH_2Cl_2 (20 mL) for three times. The aqueous layer was combined, dried over anhydrous $MgSO_4$, and evaporated. The residue was dissolved in dry 20 mL CH_2Cl_2 and cooled to 0 °C. Then, pyridine (1.16 mL, 13.1 mmol) was added dropwise. After stirring for 5 min, Tf_2O (1.0 M in CH_2Cl_2, 10 mL, 10 mmol) was added dropwise at 0 °C under argon atmosphere. The mixture solution was allowed to warm to room temperature and stirred for 2 h. The reaction mixture was poured into 100 mL of water and extracted with CH_2Cl_2 (30 mL) for three times. The organic layers were combined, washed with brine, dried over $MgSO_4$, and evaporated. The residue was purified by silica gel column chromatography (eluent: hexane: CH_2Cl_2 = 5:1) to afford the title compound as white solid (2.87 g, 87% yield). ^1H NMR (500 MHz, $CDCl_3$) δ 7.64 (dd, J = 8.0, 1.8 Hz, 1H), 7.58 - 7.47 (m, 8H), 7.46 - 7.41 (m, 2H), 1.38 (s, 9H), 1.37 (s, 9H). ^{13}C NMR (126 MHz, $CDCl_3$) δ 151.46, 147.25, 142.09, 135.98, 133.64, 132.39, 132.10, 129.02, 126.75, 126.06, 125.45, 120.41, 34.66, 31.31. HRMS (ESI, Positive): *m/z* Calcd. $[C_{27}H_{29}F_3O_3S]^+$: 489.1790 $[M]^+$, found: 489.1839.

4,4''-di-*tert*-butyl-2'-(4',4',5',5'-tetramethyl-1',3',2'-dioxaborolan-2'-yl)-1,1':4',1''-terphenyl (**8**)

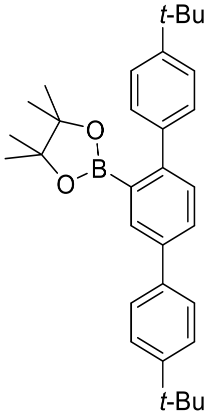

A 250-mL flask was charged with compound **S3** (2.60 g, 5.30 mmol), potassium acetate (KOAc) (1.56 g, 15.9 mmol), [1,1'-bis(diphenylphosphino)ferrocene]dichloropalladium(II) (Pd(dppf)Cl_2) (193 mg, 0.265 mmol), bis (pinacolato)diboron (2.02 g, 7.95 mmol), and dry DMF (30 mL). After the solution was degassed by argon bubbling for 10 min, the reaction mixture was heated at 90 °C for 20 h under argon atmosphere. After the reaction was completed, the mixture was poured into 200 mL of water and extracted with diethyl ether (40 mL) for three times. The organic phases were combined, dried over $MgSO_4$, and evaporated. The residue was purified by silica gel column chromatography (eluent: hexane: EtOAc = 20 :1) to give the title compound (1.78 g, 72% yield) as white solid. ^{1}H NMR (500 MHz, $CDCl_3$) δ 7.92 - 6.88 (m, 11H), 1.34 - 1.23 (m, 18H), 1.15 (m, 12H). ^{13}C NMR (126 MHz, $CDCl_3$) δ 150.09, 149.79, 148.86, 146.03, 144.24, 141.92, 139.92, 139.21, 138.71, 138.09, 132.96, 131.03, 129.48, 129.14, 128.69, 127.32, 126.83, 126.36, 125.63, 124.78, 83.76, 34.49, 31.37, 24.60, 22.66, 14.14. HRMS (ESI, Positive): *m/z* Calcd. For $[C_{32}H_{42}BO_2]^+$: 469.3272 [M+H]$^+$, found: 469.3253.

2-(4,4''-di-*tert*-butyl-[1,1':4',1''-terphenyl]-2'-yl)-10,10'-dimesityl-9,9'-bianthracene (**9**)

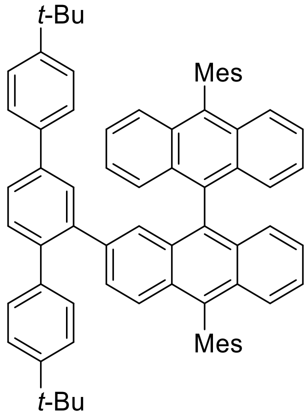

A 50-mL Schlenk tube was charged with compound **7** (200 mg, 0.271 mmol), compound **8** (190 mg, 0.407 mmol), $Pd_2(dba)_3$ (12.4 mg, 13.5 μmol), SPhos (11.1 mg, 27.1 μmol), K_2CO_3 (2.0 M, 2.0 mL), and toluene (5.0 mL). After degassing by argon bubbling for 15 min, the reaction mixture was heated at 96 °C under argon atmosphere and stirred overnight. After cooling to room temperature, the resulting mixture poured into 50 mL of water and was extracted with CH_2Cl_2 (20 mL) for three times. The separated organic phases were combined, washed with brine, dried over $MgSO_4$, and evaporated. The residue was purified by silica gel column chromatography (eluent: hexane: CH_2Cl_2 = 10: 1) to give the title compound (205 mg, 81% yield) as the light yellow solid. ^{1}H NMR (400 MHz, CD_2Cl_2) δ 7.58 - 7.46 (m, 3H), 7.42 - 7.34 (m, 2H), 7.30 - 7.09 (m, 16H), 7.05 (s, 6H), 6.86 (d, J = 8.4 Hz, 2H), 6.73 (dd, J = 9.0, 1.7 Hz, 1H), 2.55 - 2.18 (m, 6H), 1.98 - 1.67 (m, 9H), 1.26 (d, J = 8.8 Hz, 9H), 1.17 (d, J = 4.6 Hz, 9H). ^{13}C NMR (101 MHz, CD_2Cl_2) δ 150.43, 150.32, 149.60, 140.67, 139.85, 139.62, 139.55, 139.41, 139.05, 138.51, 137.79, 137.66, 137.51, 137.46, 137.32, 137.09, 136.70, 136.28, 134.66, 134.61, 132.93, 132.87, 131.75, 131.70, 131.55, 130.74, 129.92, 129.78, 129.65, 129.34, 128.78, 128.57, 128.39, 128.25, 127.98, 127.71, 127.31, 127.16, 127.05, 126.38, 126.28, 125.64, 125.49, 125.40, 125.01, 124.66, 124.52, 34.36, 31.58, 31.05, 22.65, 20.94, 19.83, 19.57, 13.87. HRMS (MALDI-TOF): *m/z* Calcd. For $[C_{72}H_{66}]^+$: 930.5165 [M]$^+$, found: 930.5184.

12,19-di-*tert*-butyl-9,22-dimesityldibenzo[*a*,*m*]dinaphtho[3,2,1-*ef*:1',2',3'-*hi*]coronene (DBDNC **1**)

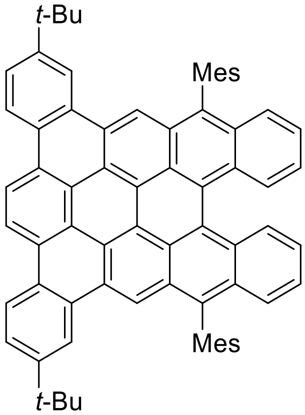

A 30-mL Schlenk tube was charged with compound **9** (30 mg, 0.032 mmol), 2,3-dichloro-5,6-dicyano-1,4-benzoquinone (DDQ) (0.11 g, 0.48 mmol), scandium (III) triflate (0.25 g, 0.58 mmol), and trifluoromethanesulfonic acid (TfOH) (0.20 mL) in 1,2-dichlorobenzene (6.0 mL). The reaction mixture was subjected to freeze-pump-thaw cycles (3 times) and heated at 140 ºC for 2 h under argon atmosphere. Then, after the resulting mixture was cooled to room temperature, dimethylamine (1.0 mL) was added to the reaction mixture and stirred for 10 minutes. The reaction mixture was poured into water/THF (30/30 mL). The organic layer was separated, and the aqueous layer was extracted with THF (2 × 30 mL). The separated organic phases were combined, washed with brine, dried over $MgSO_4$, and evaporated. The residue was purified by silica gel column chromatography (eluent: toluene: hexane = 5:1) to give the crude product. By using preparative high-performance liquid chromatography (HPLC) on a COSMOSIL Buckyprep 20ID × 250 mm column with toluene/isopropyl alcohol (1:1) as eluent, the title compound was completely separated as dark blue solid (3.0 mg, 10% yield). ^{1}H NMR (500 MHz, THF-d_8:CS_2 = 1:1) δ 9.24 (s, 2H), 9.07 (s, 2H), 8.79 (d, J = 8.5 Hz, 2H), 8.44 (d, J = 2.1 Hz, 2H), 8.33 (d, J = 8.6 Hz, 2H), 8.02 (dd, J = 8.6, 1.3 Hz, 2H), 7.75 (dd, J = 8.5, 2.0 Hz, 2H), 7.55 (ddd, J = 8.6, 6.4, 1.1 Hz, 2H), 7.38 (ddd, J = 8.7, 6.4, 1.3 Hz, 2H), 7.30 (s, 2H), 7.25 (s, 2H), 2.55 (s, 6H), 2.07 (s, 6H), 1.76 (s, 6H), 1.43 (s, 18H). ^{13}C NMR (126 MHz, THF-d_8:CS_2 = 1:1) δ 150.29, 138.30, 137.97, 137.63, 136.20, 135.14, 130.48, 130.25, 129.47, 129.19, 128.88, 128.79, 128.71, 128.38, 127.17, 126.32, 126.04, 125.15, 124.98, 124.72, 123.97, 123.21, 121.83, 120.55, 119.90, 118.33, 34.83, 30.85, 30.01, 21.01, 20.42, 19.81. HRMS (MALDI-TOF): *m/z* Calcd. For $[C_{72}H_{58}]^+$: 922.4539 $[M]^+$, found: 922.4577.

3. NMR and MS spectra

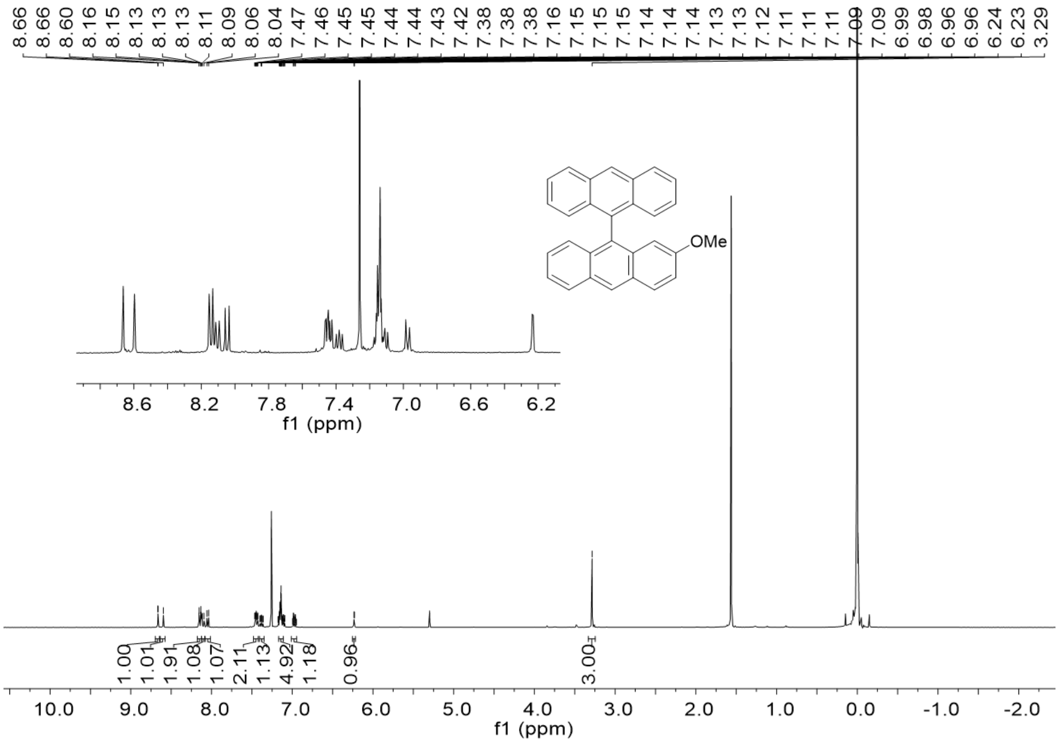

Figure S1. ^{1}H NMR spectrum of compound **4** in $CDCl_3$ (400 MHz, 298 K).

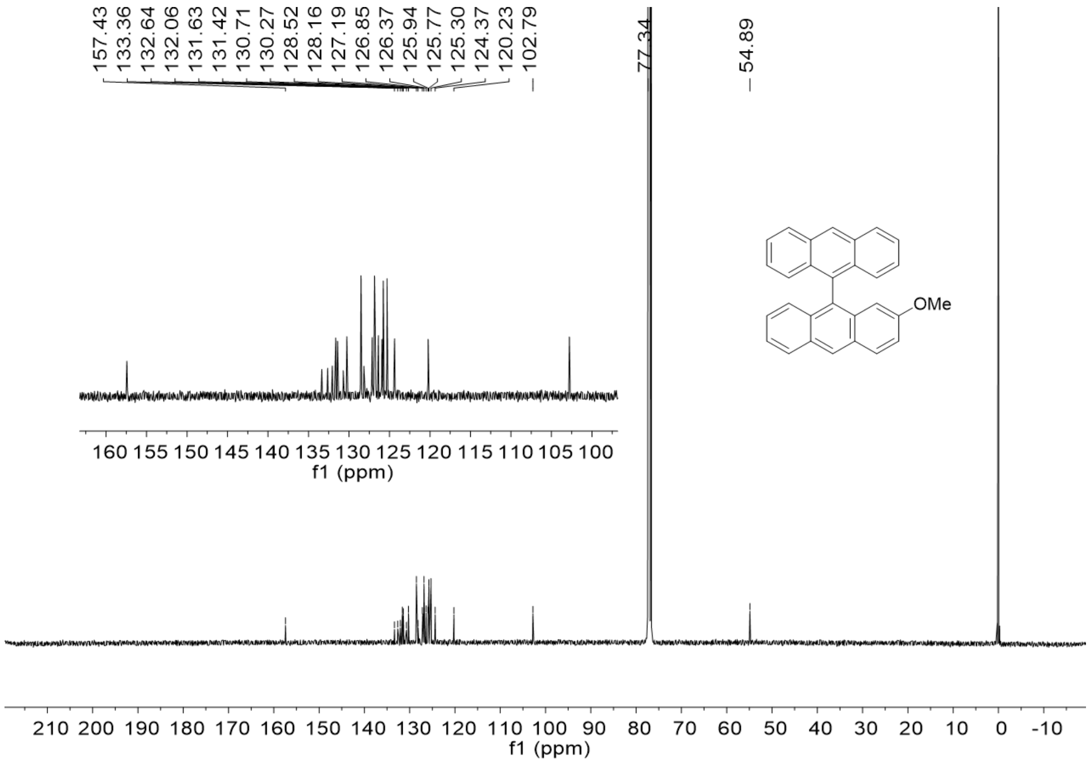

Figure S2. ^{13}C NMR spectrum of compound **4** in $CDCl_3$ (101 MHz, 298 K).

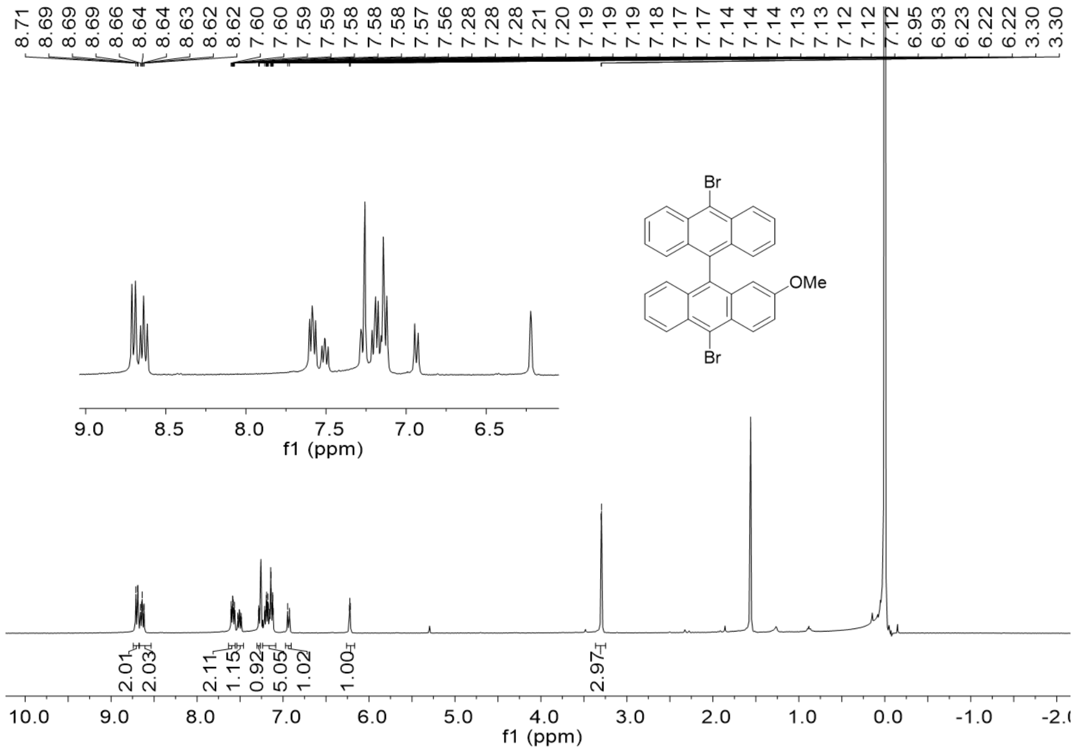

Figure S3. ^{1}H NMR spectrum of compound **5** in $CDCl_3$ (400 MHz, 298 K).

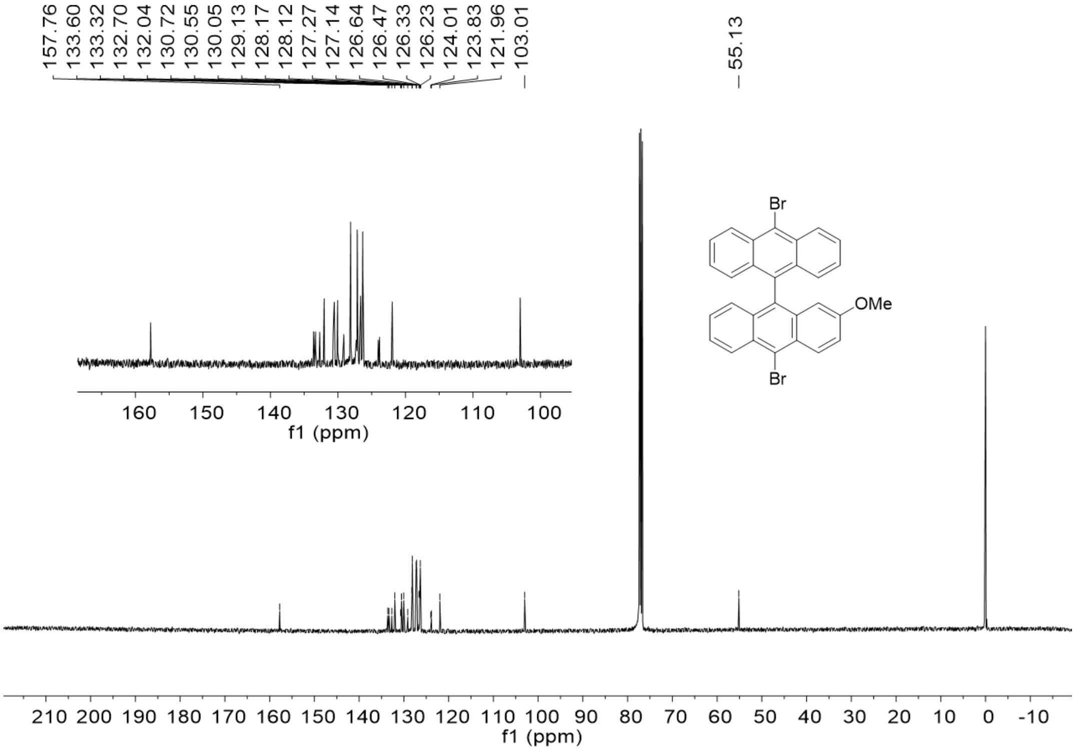

Figure S4. ^{13}C NMR spectrum of compound **5** in $CDCl_3$ (101 MHz, 298 K).

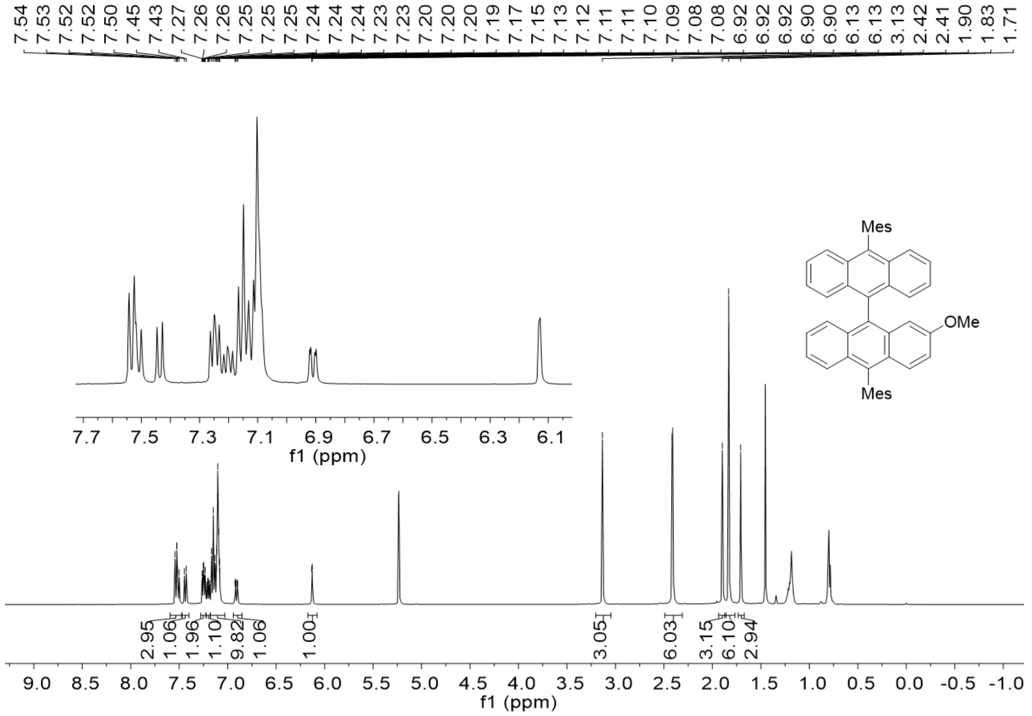

Figure S5. ^{1}H NMR spectrum of compound **6** in CD_2Cl_2 (500 MHz, 298 K).

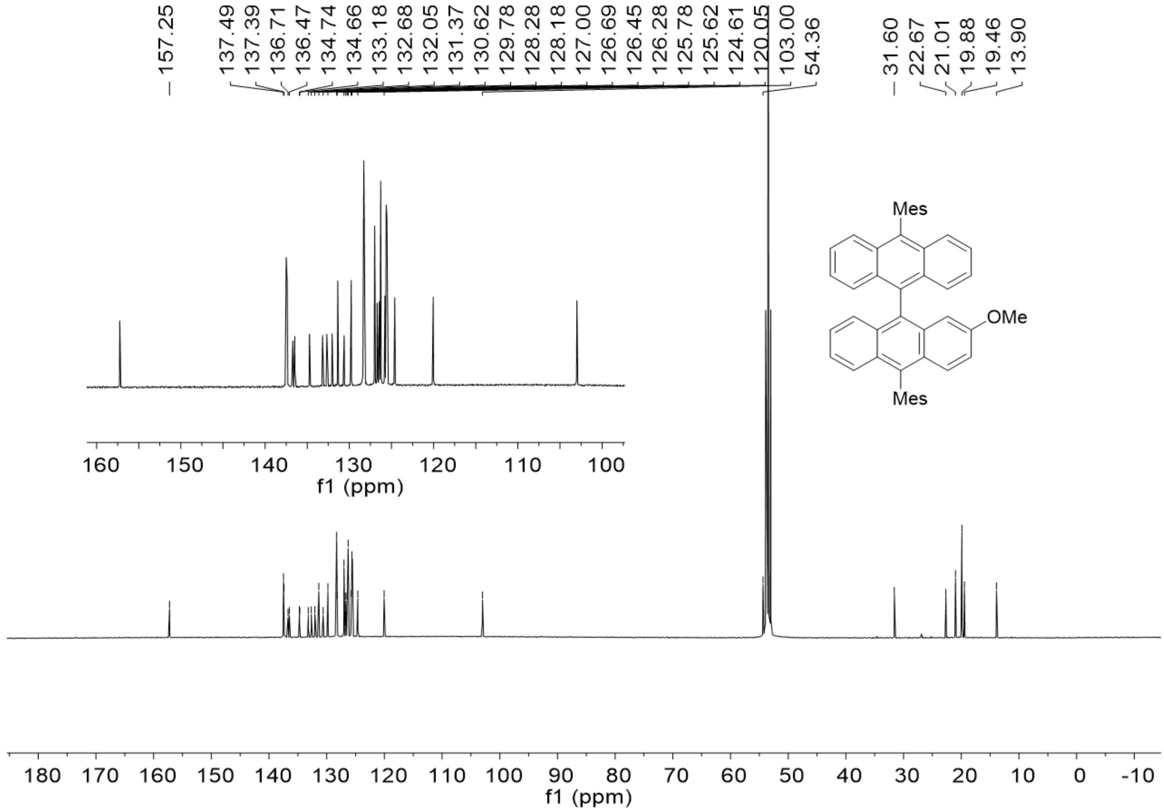

Figure S6. ^{13}C NMR spectrum of compound **6** in CD_2Cl_2 (125 MHz, 298 K).

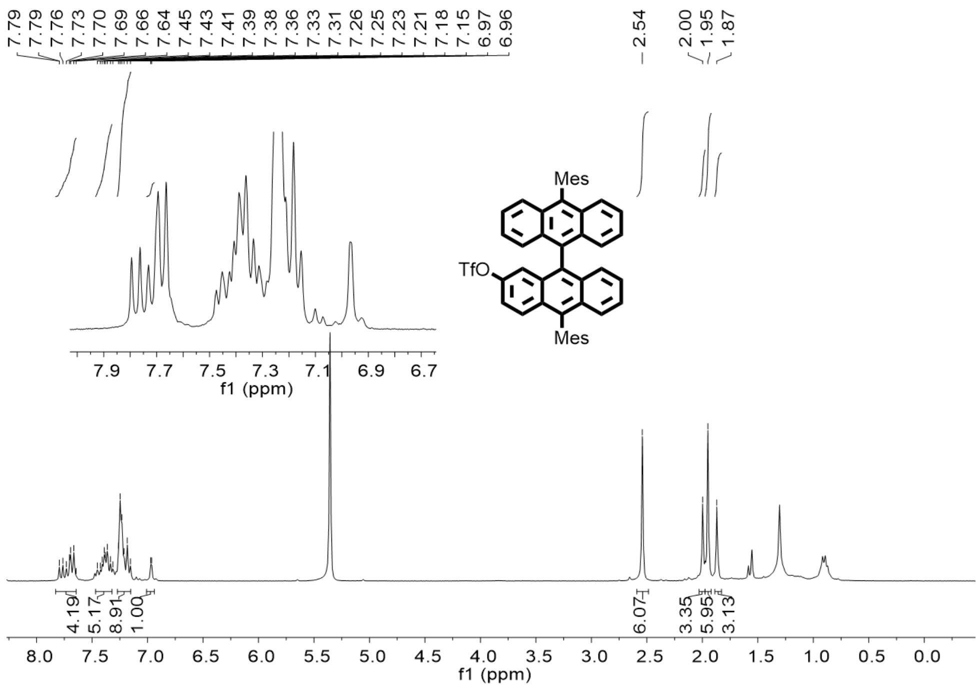

Figure S7. ^{1}H NMR spectrum of compound **7** in CD_2Cl_2 (300 MHz, 298 K).

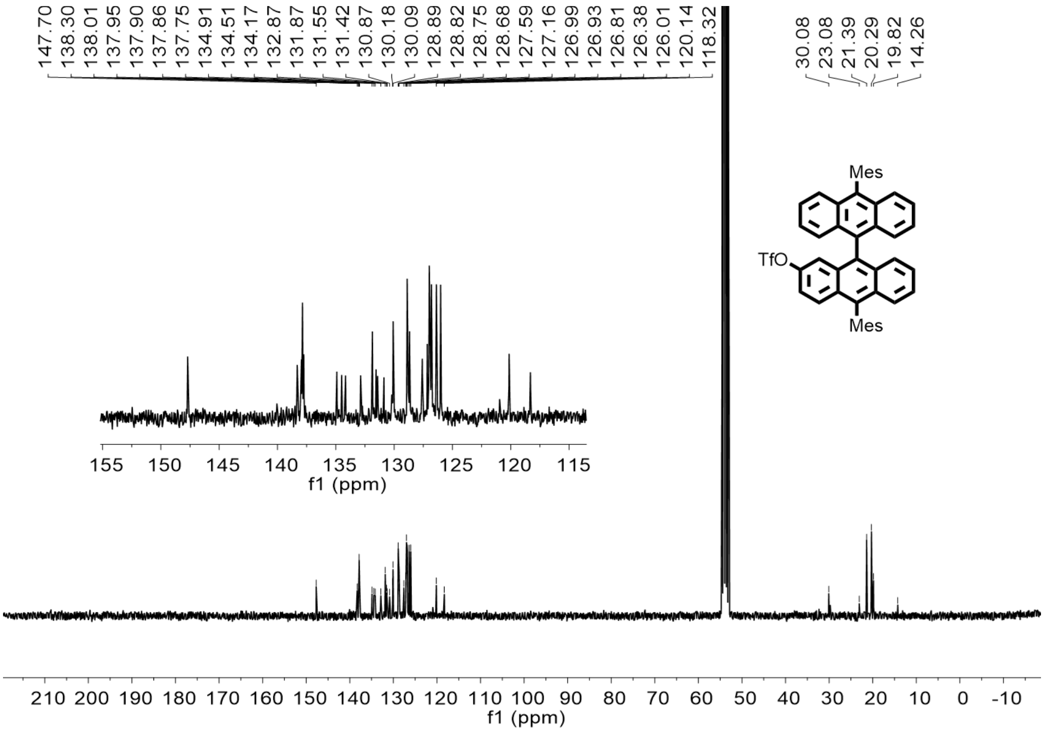

Figure S8. ^{13}C NMR spectrum of compound **7** in CD_2Cl_2 (75 MHz, 298 K).

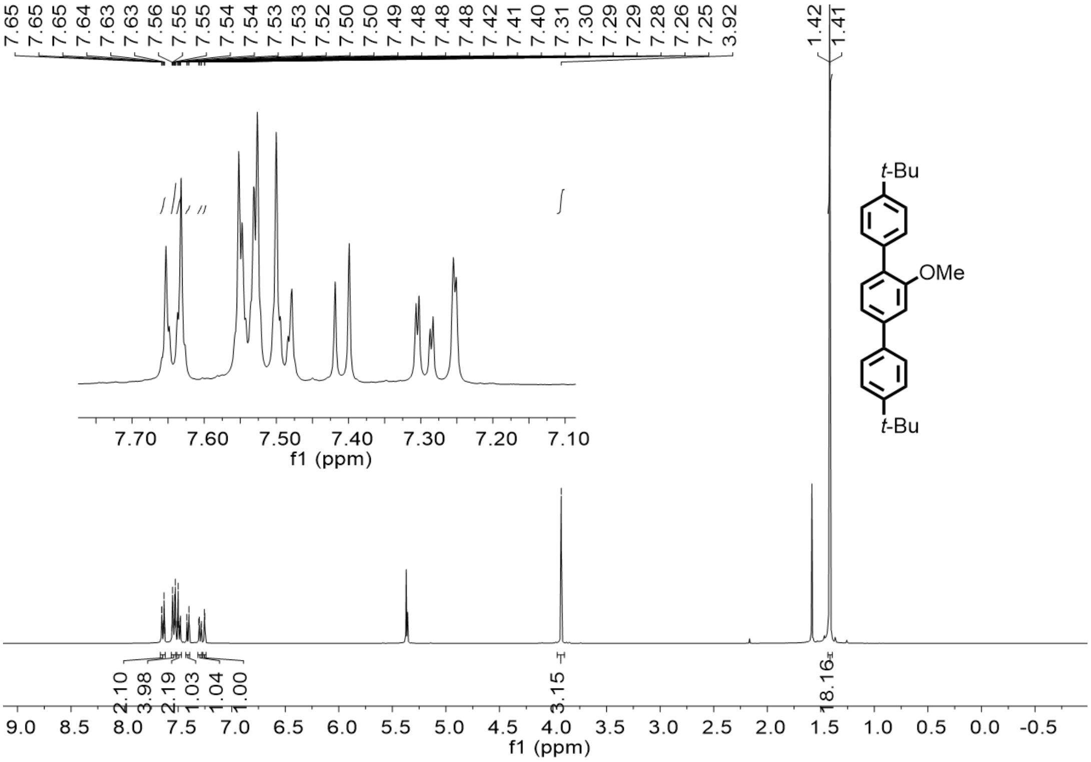

Figure S9. ^{1}H NMR spectrum of compound **S1** in CD_2Cl_2 (400 MHz, 298 K).

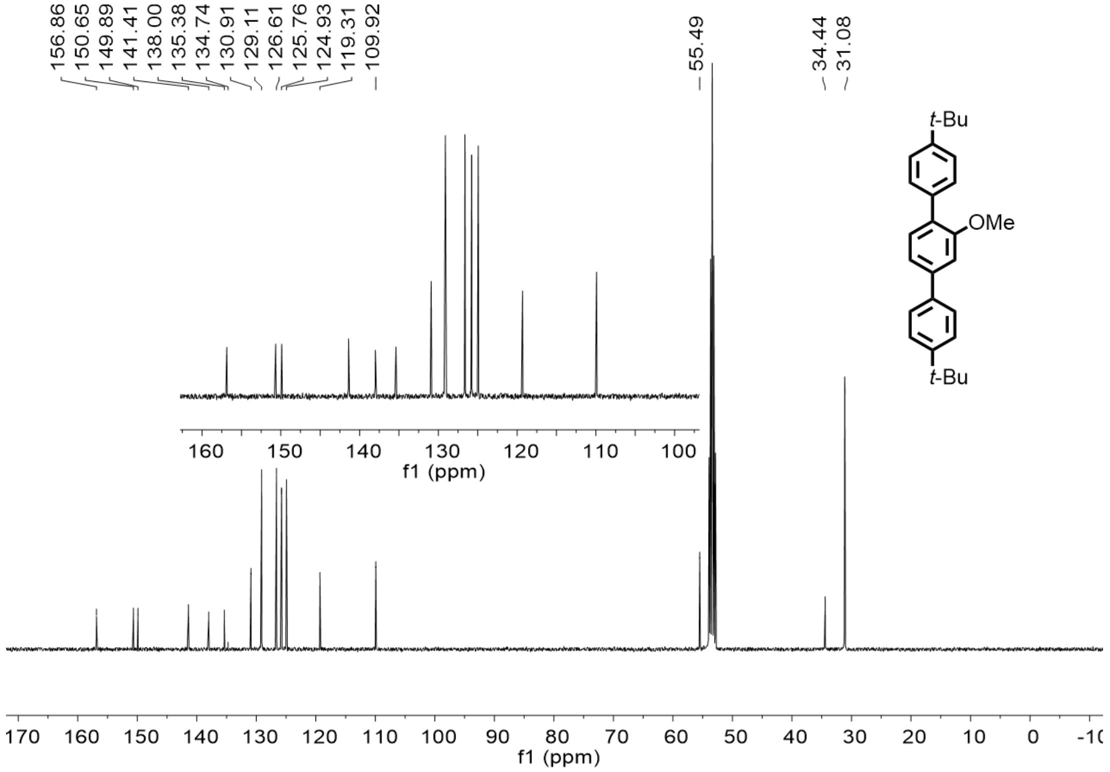

Figure S10. ^{13}C NMR spectrum of compound **S1** in CD_2Cl_2 (101 MHz, 298 K).

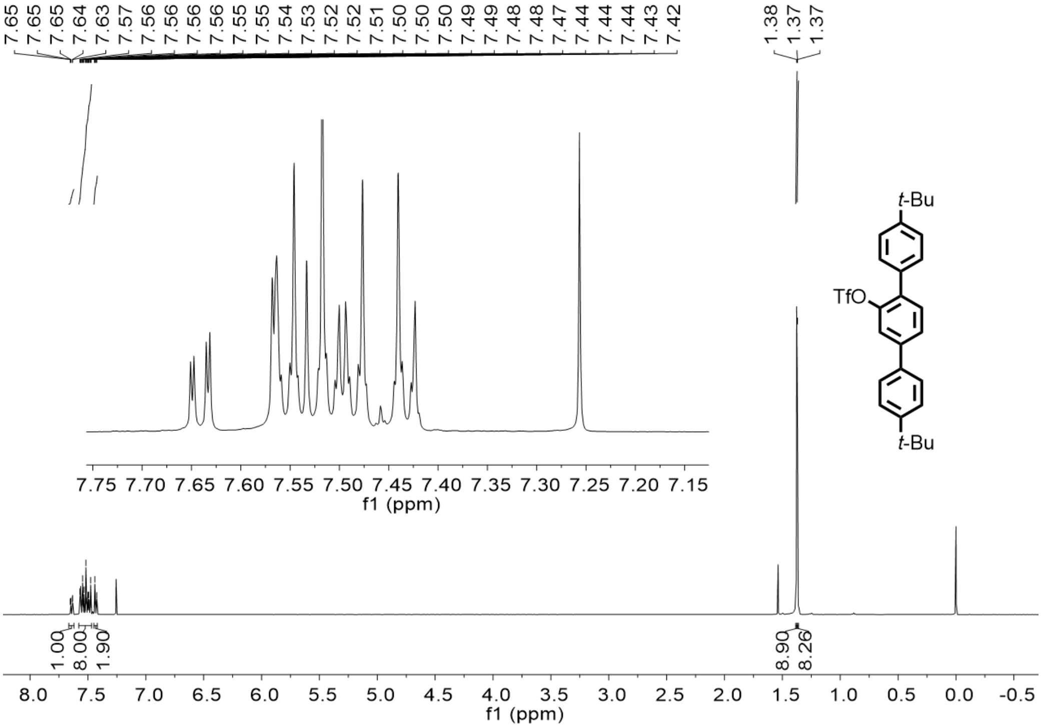

Figure S11. ^{1}H NMR spectrum of compound **S2** in CD_3Cl (500 MHz, 298 K).

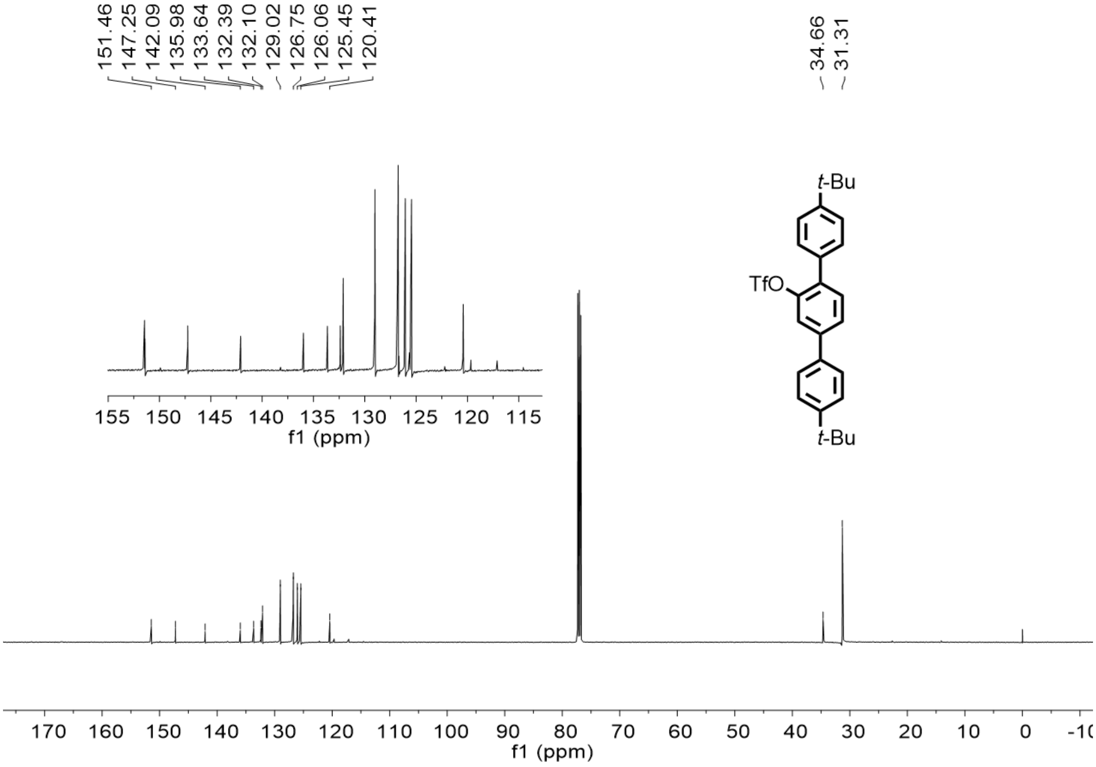

Figure S12. ^{13}C NMR spectrum of compound **S2** in CD_3Cl (126 MHz, 298 K).

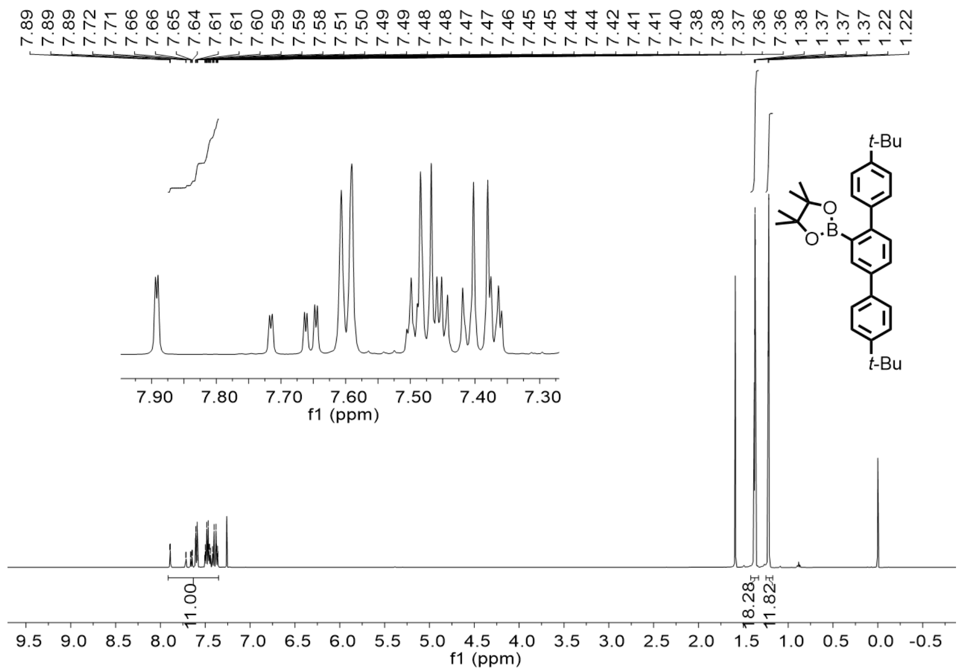

Figure S13. ^{1}H NMR spectrum of compound **8** in CD_3Cl (500 MHz, 298 K).

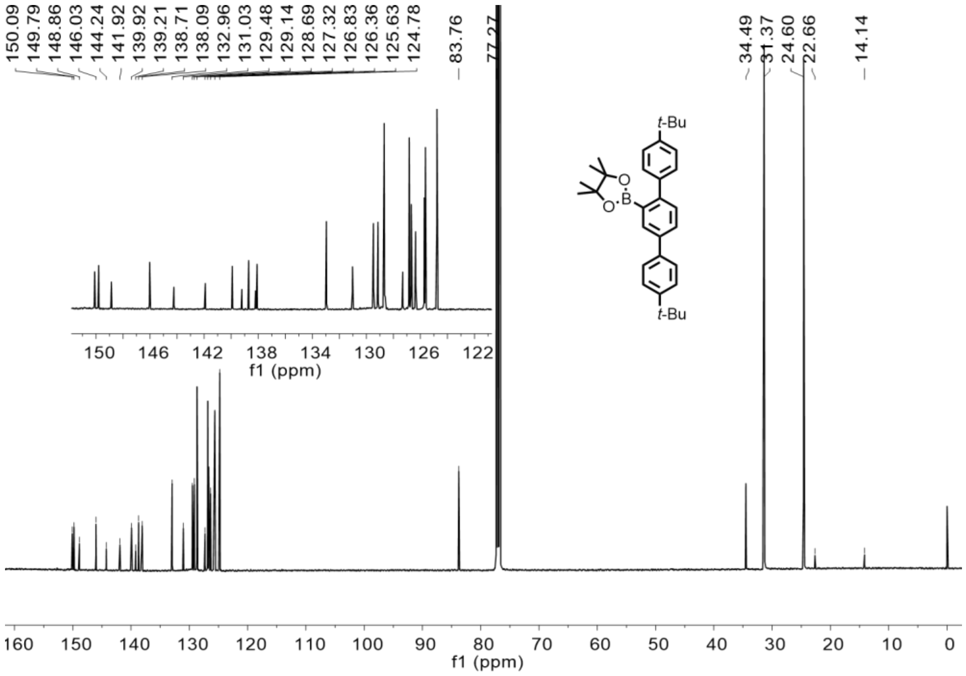

Figure S14. ^{13}C NMR spectrum of compound **8** in CD_3Cl (126 MHz, 298 K).

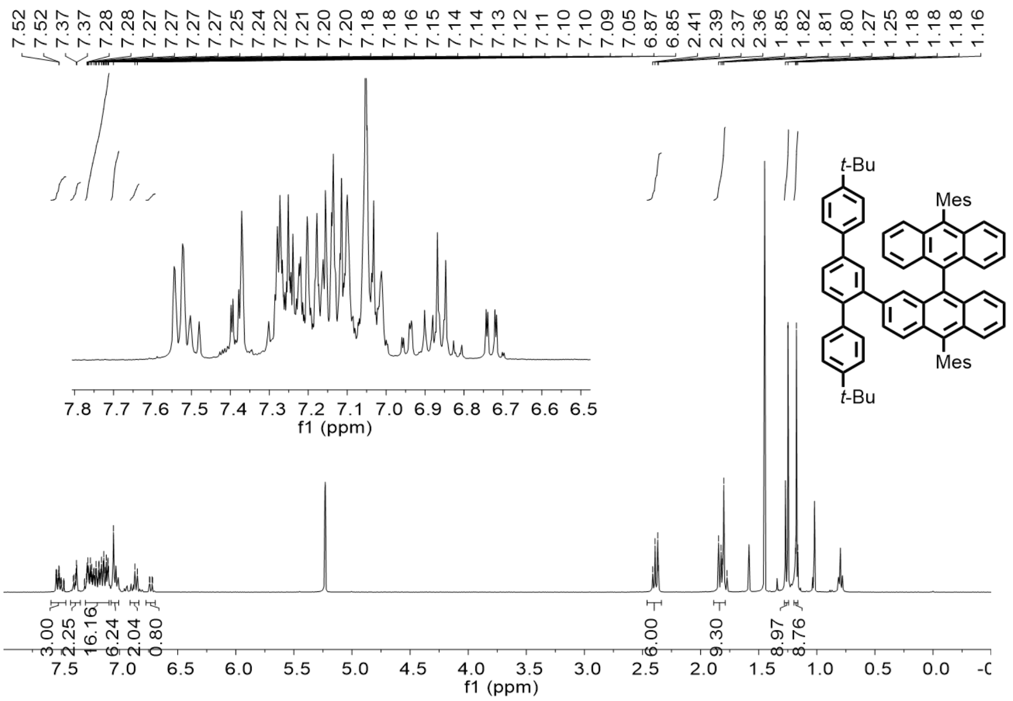

Figure S15. ^{1}H NMR spectrum of compound **9** in CD_2Cl_2 (400 MHz, 298 K).

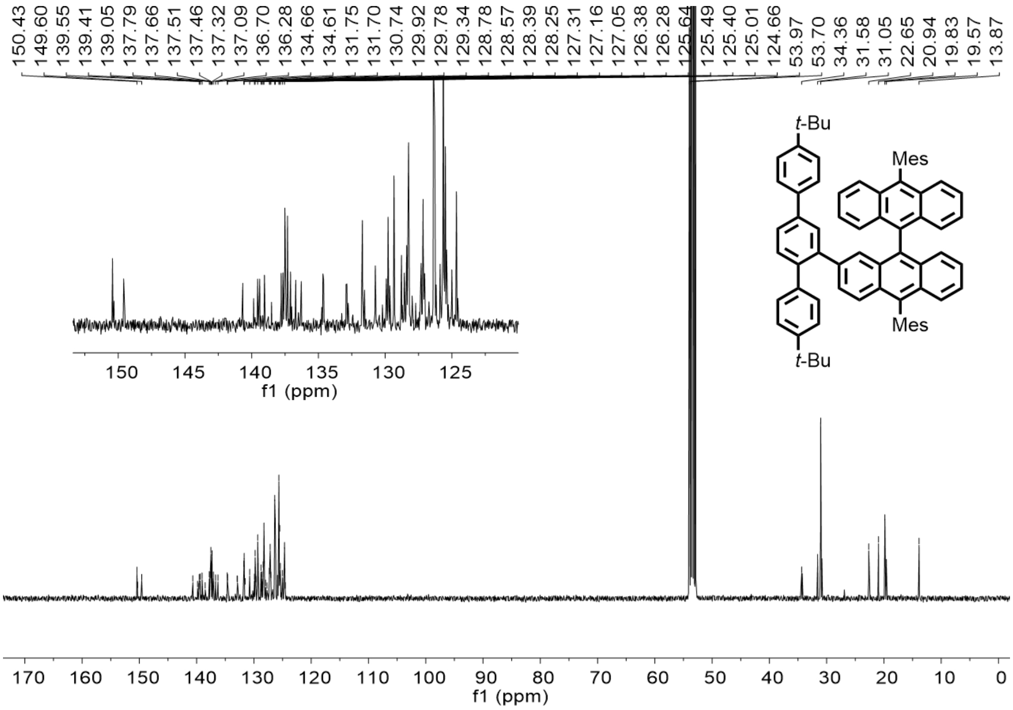

Figure S16. ^{13}C NMR spectrum of compound **9** in CD_2Cl_2 (101 MHz, 298 K).

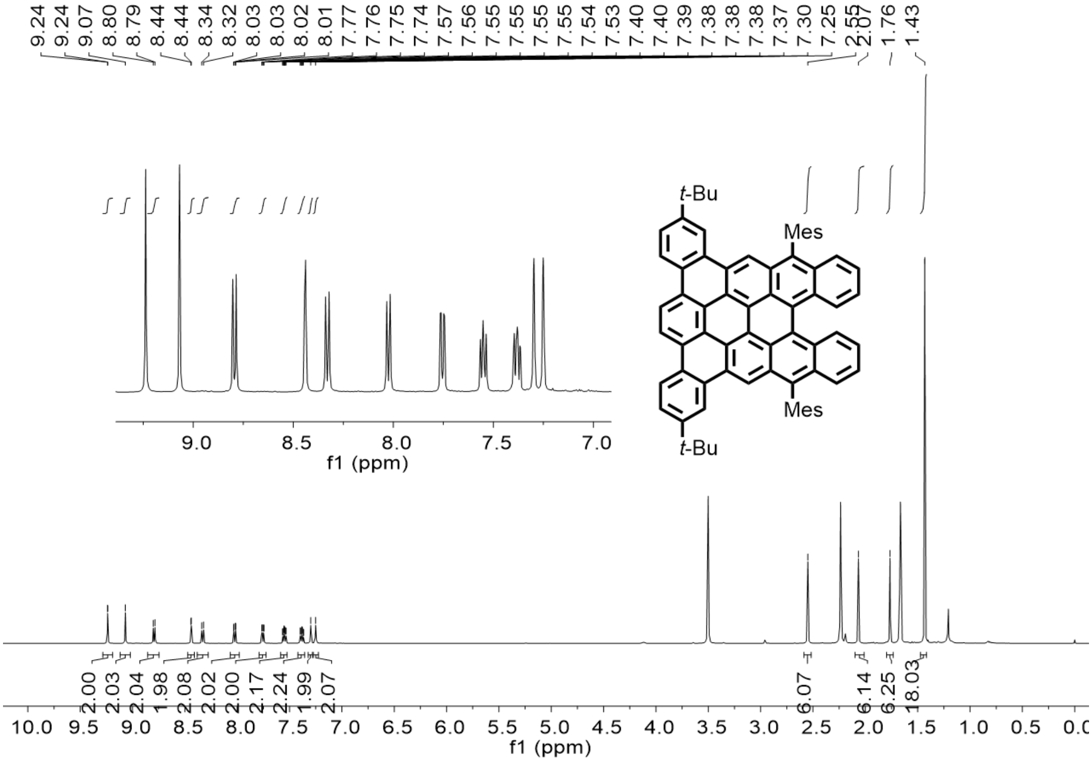

Figure S17. ^{1}H NMR spectrum of compound **1** in THF-*d*8:CS_2 = 1:1 (500 MHz, 298 K).

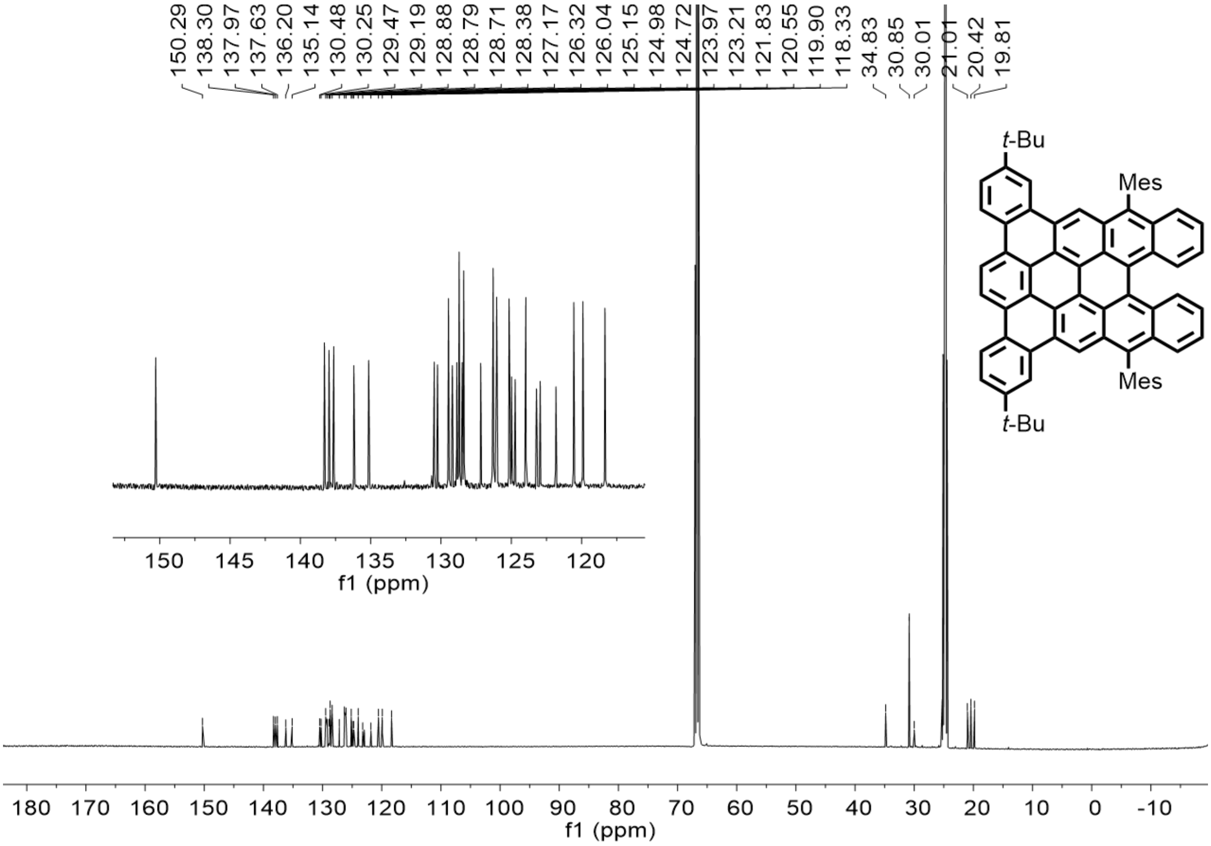

Figure S18. ^{13}C NMR spectrum of compound **1** in THF-d_8:CS_2 = 1:1 (126 MHz, 298 K).

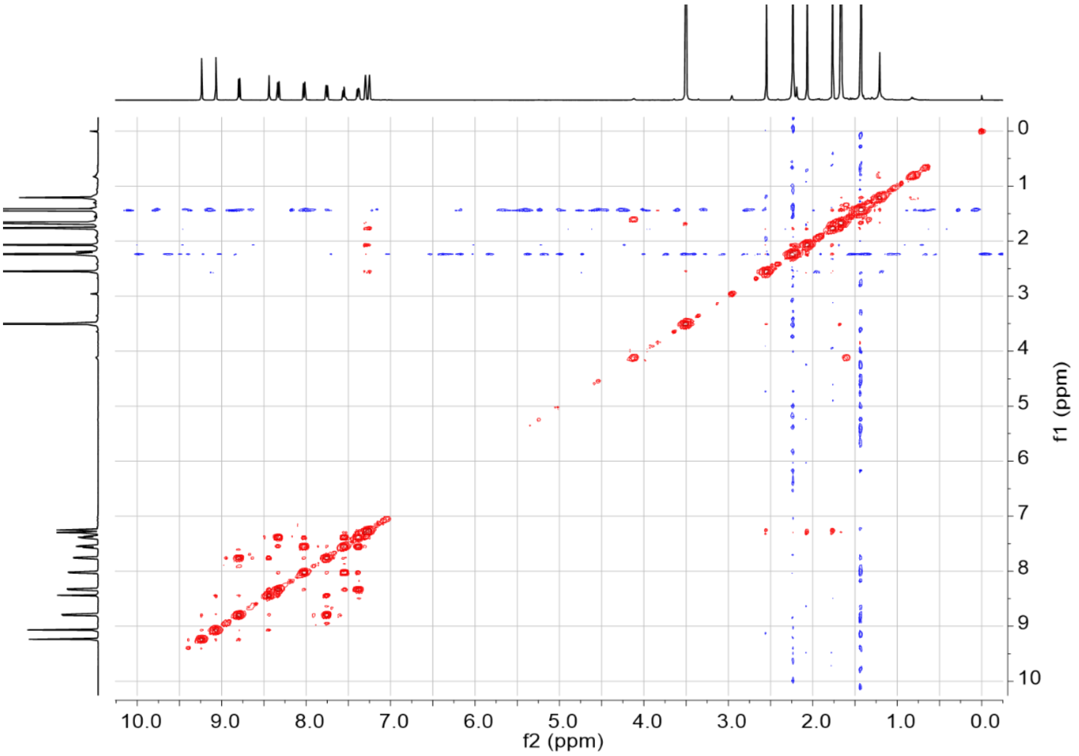

Figure S19. ^{1}H-^{1}H COSY spectrum of compound **1** in THF-d_8:CS_2 = 1:1 (500 MHz, 298 K).

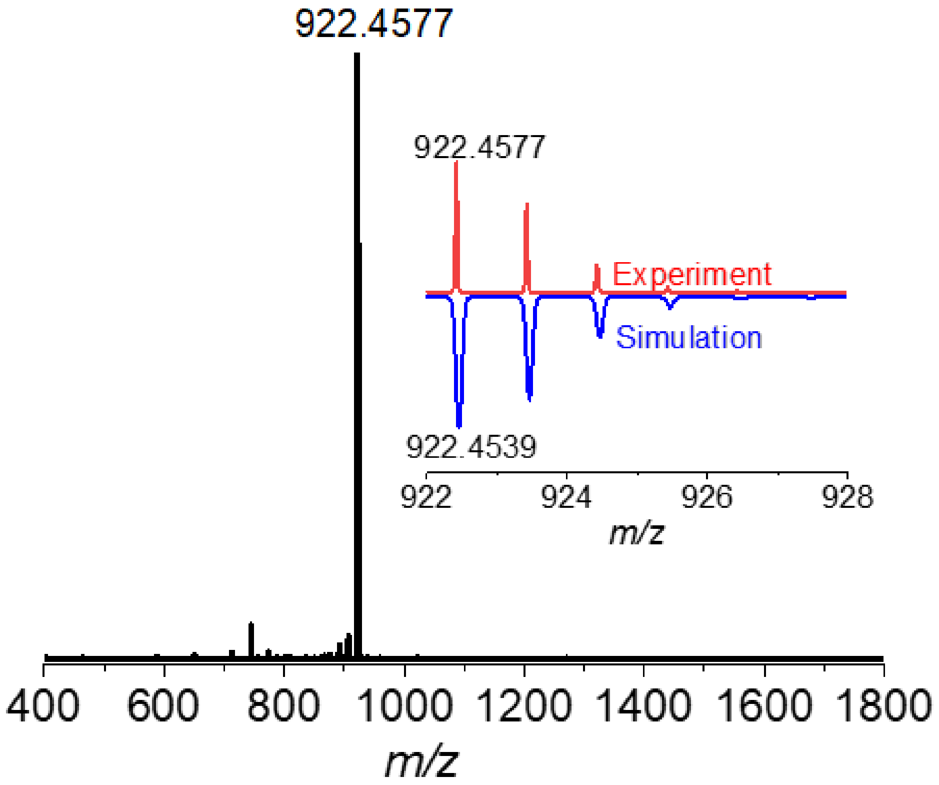

Figure S20. High-resolution MALDI-TOF MS spectrum of DBDNC **1** after purification. Inset: the corresponding experimental and simulated isotopic distributions. DBDNC 1 exhibited a single peak at *m/z* = 922.4577, consistent with its expected molecular mass of 922.4539. And the observed isotopic distribution of DBDNC **1** was in perfect match with the simulated pattern.

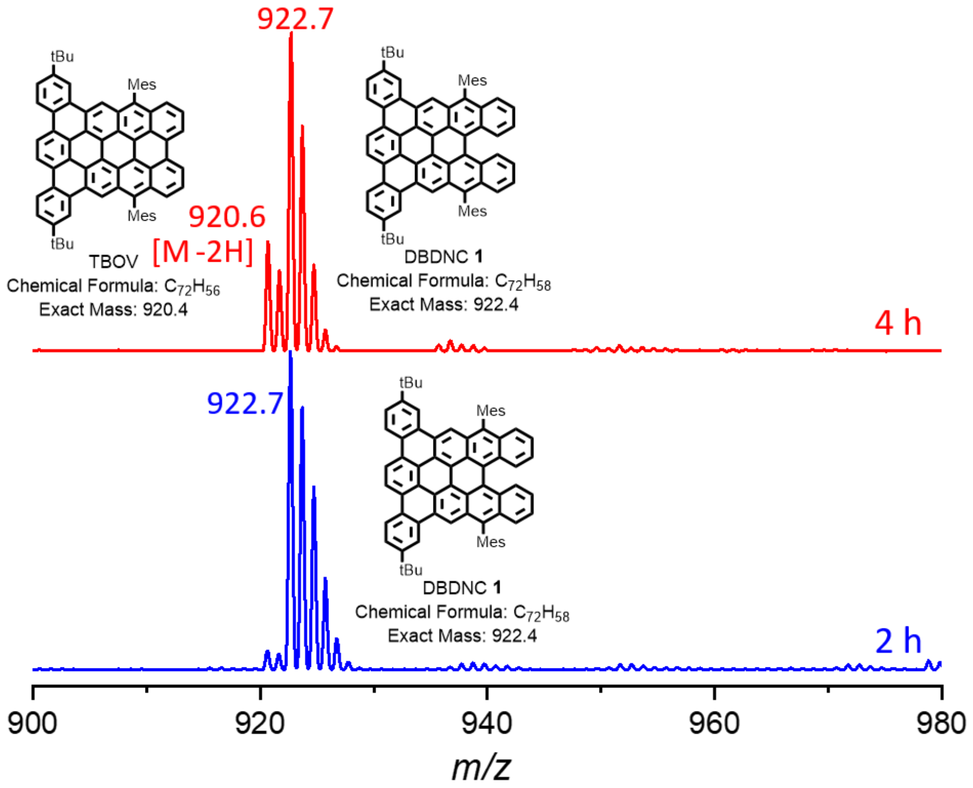

Figure S21. MALDI-TOF MS spectrum after oxidation of **9** (DDQ, $Sc(OTf)_3$,TfOH, 140 °C in 1,2-dichlorobenzene), which showed an additional signal that was tentatively assigned to TBOV.

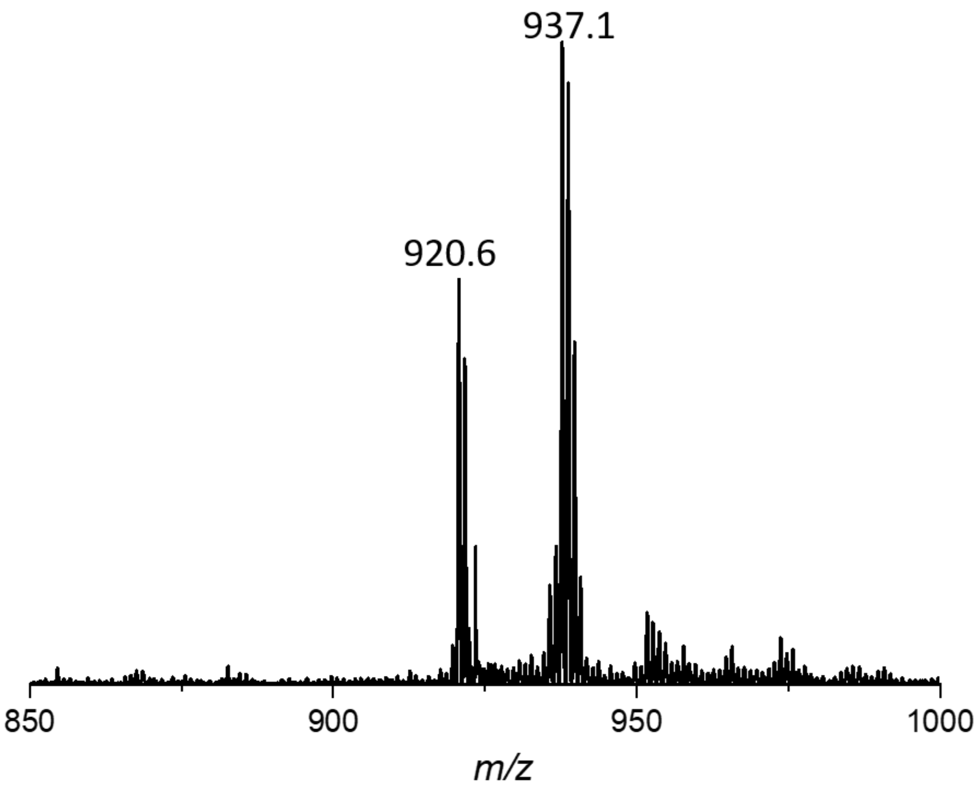

Figure S22. MALDI-TOF MS spectrum of the second product after attempted isolation, which denoting its oxidation.

4. X-ray crystallography

Experimental details: The single crystal of DBDNC **1** suitable for X-ray analysis was obtained by slow evaporation of its solution in benzene/methanol mixture, giving single metallic-dark-blue, block-shaped crystals. A suitable crystal with dimensions 0.23 × 0.14 × 0.11 mm^3 was selected and mounted on a Bruker SMART APEX3 area detector diffractometer. The crystal was kept at a steady T = 100 K during data collection. The structure was solved with the ShelXT 2018/2 (Sheldrick, 2018) solution program using iterative methods and by using Olex2 1.5 (Dolomanov et al., 2009) as the graphical interface. The model was refined with XL (Sheldrick, 2008) using full matrix least squares minimisation on F2.

The structure was deposited at the Cambridge Crystallographic Data Centre (CCDC) and the data could be obtained free of charge via www.ccdc.cam.ac.uk/structures. Crystal data for DBDNC **1** (CCDC number: 2167126).

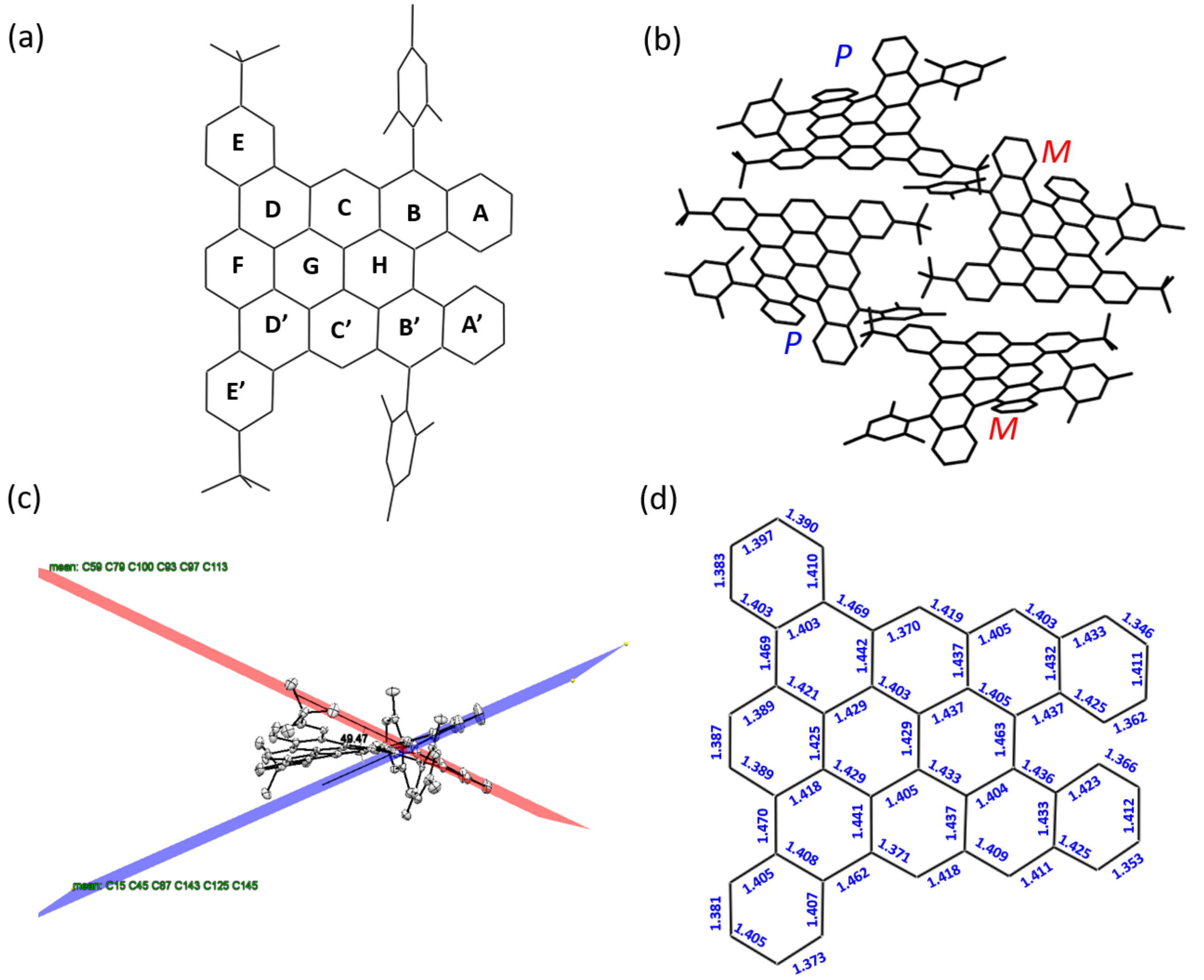

Figure S23. X-ray crystal structure of DBDNC **1**. (a) The top view of the DBDNC whose rings was marked for clarifications. The torsion angle of ring *A* and *A*' was estimated to be about 49.5°. (b) The packing mode of DBDNC **1** in unit cell, showing with two absolute configurations of (*P*)- and (*M*)- isomer. (c) The torsion angle of ring *A* and *A*' was estimated to be about 49.5°. (d) Bond lengths in the main core rings of DBDNC **1**.

Crystal Data: $C_{150}H_{122}$, M_r = 1924.47, triclinic, *P*-1 (No. 2), a = 18.1166(5) Å, b = 19.1083(9) Å, c = 19.7848(10) Å, α = 114.665(5)°, β = 95.802(3)°, γ = 90.221(3)°, *V* = 6184.2(5) Å^3, *T* = 100 K, *Z* = 2, *Z*' = 1, λ(Mo K_α) = 0.058, 88875 reflections measured, 24548 unique (Rint = 0.1176) which were used in all calculations. The final wR_2 was 0.1729 (all data) and R_1 was 0.0771 (I≥2(I)).

**Compound**	**DBDNC 1**
Formula	$C_{150}H_{122}$
$D_{calc.}$/ g cm^{-3}	1.033
μ/mm^{-1}	0.058
Formula Weight	1924.47
Colour	Metallic-dark-blue
Shape	block-shaped
Size/mm^3	0.23×0.14×0.11
T/K	100
Crystal System	triclinic
Space Group	*P*-1
a/Å	18.1166(5)
b/Å	19.1083(9)
c/Å	19.7848(10)
α /°	114.665(5)
β /°	95.802(3)
γ /°	90.221(3)
V/Å^3	6184.2(5)
Z	2
Z'	1
Wavelength/Å	0.71073
Radiation type	Mo K_α
$2\Theta_{min}$/°	1.950
$2\Theta_{max}$/°	26.372
Measured Refl's.	88875
Indep't Refl's	24548
Refl's I≥2 σ(I)	15877
R_{int}	0.1176
Parameters	1375
Restraints	0
Largest Peak	0.281
Deepest Hole	-0.243
GooF	1.020
wR_2 (all data)	0.1729
wR_2	0.1518
R_1 (all data)	0.1181
R_1	0.0771

5. DFT Calculations

DFT calculations were performed using the Gaussian 09 software package[2]. The geometry and energies were calculated at the B3LYP/6-311G(d,p) level. Time-dependent DFT (TD-DFT) calculations were performed at the CAM-B3LYP/6-311G(d,p) energy level using toluene as the solvent model (SMD). The analysis of oscillator strength *f*, absorbance was performed using a multifunctional wavefunction analyser (Multiwfn)[3].

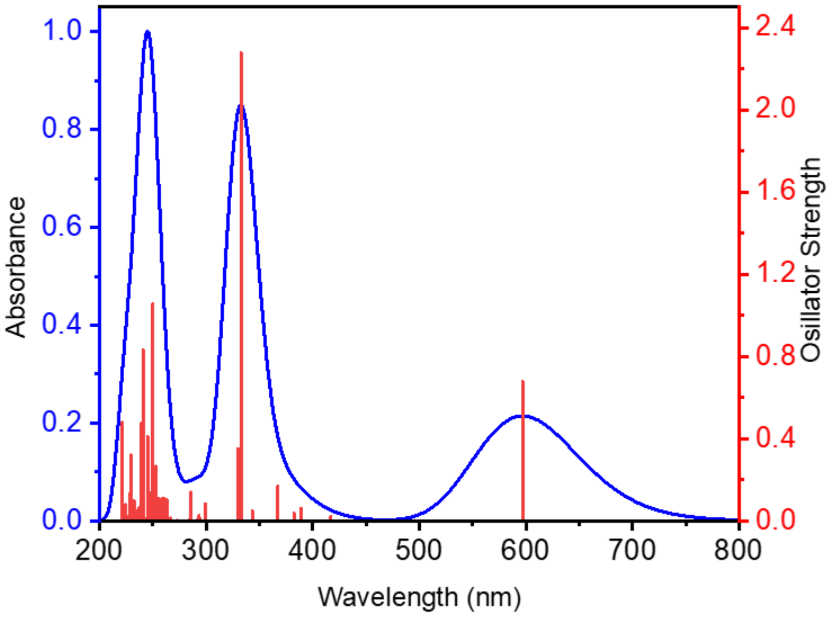

Figure S24. Simulated absorption spectrum of **1** (blue curve**)** and the oscillator strengths (red bars) by TDDFT calculations at the CAM-B3LYP/6-311G(d,p) energy level using the toluene as the solvent (model: SMD).

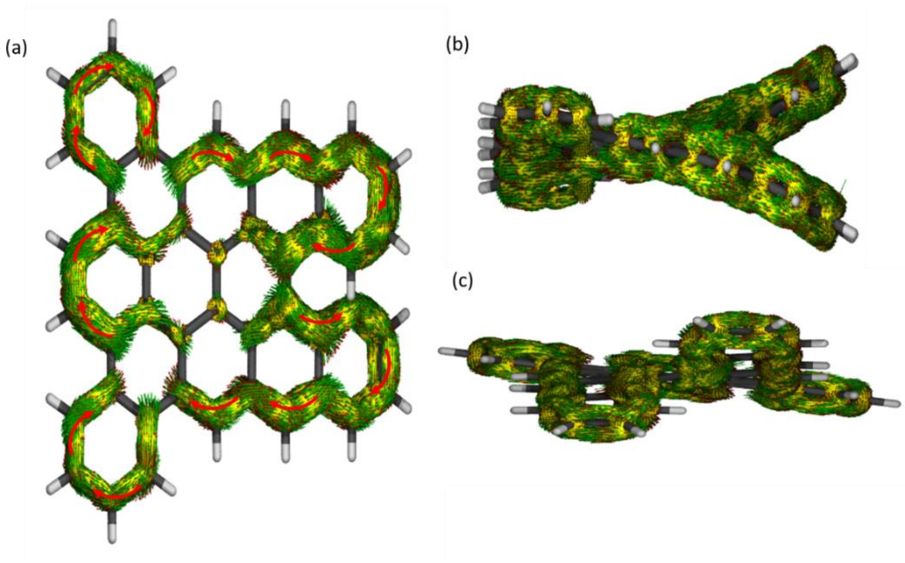

Figure S25. A π-only ACID plot [4-5] calculated for non-substituted DBDNC at the B3LYP/6-31G(d,p). (a) Top view. The ring currents under the magnetic field parallel to the z-axis are highlighted by red arrows. (b,c) Side views. Only contributions from π-electrons of the DBDNC aromatic cores are considered. The optimized structure of non-substituted DBDNC was calculated at B3LYP/6-311G(d,p) level.

Table S1. Major transitions (oscillator strength *f* over 0.10) of DBDNC **1** calculated by TDDFT.

**excited state**	**energy** (eV)	**wavelength** (nm)	**oscillator strength** (*f*)	**description**
1	2.0785	596.51	0.6769	H→ L (0.69904)
5	3.3783	367.00	0.1696	H-3 → L (0.21457)
				H-2 → L (-0.34780)
				H-1 → L (0.49948)
				H → L+1 (-0.23068)
8	3.7301	332.39	2.2772	H-4 → L (-0.10540)
				H-3 → L (0.43361)
				H-1 → L (-0.18189)
				H → L+2 (0.32855)
				H → L+3 (-0.35956)
9	3.7616	329.60	0.3500	H-4 → L (0.47284)
				H-3 → L+1 (-0.11900)
				H-2 → L (0.18387)
				H → L+2 (-0.16223)
				H → L+3 (0.33685)
				H → L+4 (0.11204)
16	4.3530	284.83	0.1387	H-12 → L (0.10280)
				H-10 → L (0.25633)
				H-3 → L+1 (0.51526)
				H-3 → L+2 (0.13817)
				H-2 → L+1 (-0.14196)
				H-1 → L+1 (0.17407)
23	4.7672	260.08	0.1098	H-10→ L (-0.12274)
				H-4 → L+2 (0.10804)
				H-4 → L (-0.22127)
				H-4 → L+4 (0.26075)
				H-3→ L+1 (0.16117)
				H-3 → L+2 (-0.19423)
				H-3 → L+3 (0.19871)
				H-2 → L+3 (0.27948)
				H-1 → L+2 (0.39591)
				H-1 → L+3 (-0.13741)
24	4.8122	257.64	0.1064	H-11→ L (-0.29342)
				H-10 → L (0.11026)
				H-9 → L (0.29140)
				H-4 → L+1 (0.31330)
				H-2→ L+1 (-0.27314)
				H-1 → L+1 (-0.14410)
				H-1 → L+4 (-0.10192)
25	4.8640	254.90	0.1071	H-12→ L (0.11780)
				H-11 → L (0.30230)
				H-9 → L+2 (-0.11452)
				H-4 → L+1 (0.13695)
				H-2→ L+1 (-0.14735)
				H-2→ L+2 (-0.13689)
				H → L+5 (-0.13271)
				H → L+6 (-0.17486)
				H → L+9 (0.31453)
26	4.9158	252.22	0.2641	H-12→ L (0.28864)
				H-9 → L+1 (-0.10216)
				H-3 → L+2 (-0.16430)
				H-3 → L+3 (0.19094)
				H-2 → L+2 (0.25374)
				H-2 → L+4 (-0.12342)
				H-1 → L+3 (0.29185)
				H→ L+9 (0.18430)
				H→ L+11 (-0.14646)
29	4.9665	249.64	1.0550	H-12→ L (-0.19079)
				H-4→ L+2 (-0.13298)
				H-3 → L+1 (0.13787)
				H-3 → L+2 (-0.27913)
				H-3 → L+3 (0.32936)
				H-2 → L+2 (-0.15092)
				H-2 → L+3 (-0.10490)
				H-1 → L+2 (-0.21667)

				H-1 → L+3 (-0.14948)
				H→ L+7 (0.10484)
31	5.0096	247.49	0.1351	H-4→ L+2 (0.21517)
				H-4→ L+3 (0.11360)
				H-4→ L+5 (0.11274)
				H-3→ L+2 (0.14198)
				H-3→ L+4 (-0.18505)
				H-2→ L+3 (0.35524)
				H-2→ L+4 (-0.15301)
				H-1→ L+2 (-0.19478)
				H-1→ L+4 (0.28165)
				H→ L+9 (-0.10006)
32	5.0646	244.81	0.4093	H-14→ L (0.15655)
				H-12→ L (-0.26676)
				H-2→ L+2 (0.23232)
				H-2→ L+5 (-0.10728)
				H-1→ L+1 (0.14729)
				H-1→ L+3 (0.27816)
				H→ L+9 (-0.27132)
				H→ L+11 (0.17319)
34	5.1495	240.77	0.8308	H-13→ L (-0.24074)
				H-11→ L (0.13929)
				H-4→ L+1 (0.31651)
				H-4→ L+2 (0.13017)
				H-4→ L+3 (-0.12272)
				H-4→ L+4 (0.20897)
				H-3→ L+5 (-0.12367)
				H-2→ L+3 (-0.16615)
				H-2→ L+4 (-0.11878)
				H-2→ L+6 (0.10614)
				H-1→ L+2 (0.10614)
				H-1→ L+4 (0.20649)
				H-1→ L+5 (0.11286)

Table S2. Cartesian coordinates of the DFT-optimized DBDNC **1**.

Symbol	X	Y	Z
C	2.868564	1.362196	0.148859
C	1.428464	1.381862	0.089064
C	0.717383	0.165938	0.014482
C	1.408382	-1.07985	-0.11411
C	2.8411	-1.06784	-0.22794
C	3.521144	0.163799	-0.02968
C	0.693733	-2.29592	-0.19448
C	1.3731	-3.43174	-0.74549
C	2.808975	-3.41567	-0.85891
C	3.539698	-2.2512	-0.52981
C	0.680193	-4.55115	-1.29072
C	1.346104	-5.62892	-1.81255
C	2.761738	-5.664	-1.8075
C	3.468585	-4.58544	-1.35091
C	-0.70878	-2.28939	0.207347
C	-1.41449	-1.06879	0.119025
C	-2.84705	-1.04614	0.23115
C	-3.5548	-2.22291	0.536849
C	-2.83296	-3.39043	0.874738
C	-1.39702	-3.41683	0.764714
C	-3.50153	-4.55264	1.372272
C	-2.80286	-5.63297	1.837377
C	-1.38707	-5.60732	1.845495
C	-0.71278	-4.53732	1.318363
C	-0.71454	0.171263	-0.01801
C	-1.41665	1.391839	-0.10106
C	-2.85677	1.381981	-0.1578
C	-3.51771	0.189725	0.026912

C	-3.57454	2.645485	-0.37605
C	3.593741	2.621094	0.362696
C	4.985185	2.635311	0.594859
C	5.706856	3.802891	0.792499
C	4.985556	5.012427	0.760242
C	3.622801	5.027677	0.552501
C	2.887267	3.842299	0.350602
C	-2.85666	3.863991	-0.38042
C	-3.5822	5.049616	-0.58959
C	-4.95149	5.047942	-0.78619
C	-5.68013	3.848247	-0.79656
C	-4.96265	2.673548	-0.59436
C	1.436757	3.853051	0.149901
C	-1.40459	3.863279	-0.18556
C	0.70935	5.040283	0.058887
C	-0.66689	5.045037	-0.1086
C	-0.70113	2.629646	-0.08542
C	0.722573	2.624475	0.061668
C	7.222762	3.820299	1.049602
C	7.836197	2.408381	1.052465
C	7.919107	4.645728	-0.05772
C	7.502338	4.469418	2.425325
C	-7.20134	3.793622	-1.02547
C	-7.80932	5.192776	-1.23498
C	-7.50582	2.944603	-2.28171
C	-7.88366	3.150889	0.204771
C	5.038437	-2.25831	-0.5735
C	5.770958	-2.60964	0.578115
C	7.165763	-2.61738	0.522138
C	7.859592	-2.28587	-0.64279
C	7.116262	-1.94382	-1.7717
C	5.718554	-1.9237	-1.76008
C	4.964522	-1.5528	-3.01619
C	5.06845	-2.98487	1.862423
C	9.369548	-2.27917	-0.66932
C	-5.05376	-2.21927	0.572442
C	-5.73892	-1.8703	1.75201
C	-7.13685	-1.87711	1.754006
C	-7.87564	-2.22024	0.62229
C	-7.17692	-2.56695	-0.53524
C	-5.78165	-2.57245	-0.58157
C	-5.07405	-2.96354	-1.85834
C	-4.99002	-1.4979	3.01073
C	-9.38563	-2.19857	0.638444
H	4.599904	0.133771	-0.06433
H	-0.39989	-4.53683	-1.31166
H	0.786332	-6.45336	-2.23932
H	3.282866	-6.53224	-2.19514
H	4.550597	-4.58763	-1.38791
H	-4.58365	-4.54743	1.406685
H	-3.33065	-6.49524	2.229318
H	-0.83381	-6.43284	2.278629
H	0.36737	-4.53003	1.341201
H	-4.59664	0.167669	0.061217
H	5.50599	1.691186	0.636864
H	5.497714	5.955328	0.912465
H	3.11715	5.983921	0.558867
H	-3.06892	6.001621	-0.61103
H	-5.45026	5.994892	-0.94318
H	-5.49905	1.735132	-0.62065

H	1.215616	5.9944	0.111577
H	-1.16532	6.002657	-0.17163
H	8.911873	2.478372	1.235172
H	7.409187	1.780152	1.838745
H	7.69694	1.899475	0.094913
H	8.999745	4.67299	0.112982
H	7.56019	5.677109	-0.08356
H	7.740861	4.20491	-1.04225
H	7.026875	3.899548	3.228162
H	8.578914	4.498199	2.619385
H	7.127571	5.494314	2.475826
H	-8.88784	5.103993	-1.39059
H	-7.65389	5.838646	-0.36652
H	-7.39002	5.691279	-2.11317
H	-8.58513	2.905007	-2.45901
H	-7.14821	1.918125	-2.17559
H	-7.03096	3.374664	-3.16773
H	-7.68574	3.733146	1.108869
H	-8.96743	3.108263	0.057809
H	-7.53001	2.132327	0.37943
H	7.723677	-2.89282	1.412608
H	7.634911	-1.68981	-2.69147
H	5.65594	-1.33062	-3.83103
H	4.306867	-2.36369	-3.34187
H	4.332476	-0.67403	-2.86075
H	4.441829	-3.87171	1.729439
H	5.79129	-3.19841	2.651992
H	4.412485	-2.18218	2.210679
H	9.781512	-3.12316	-0.10994
H	9.75014	-2.3325	-1.69153
H	9.767437	-1.36447	-0.21654
H	-7.65937	-1.61184	2.668364
H	-7.73118	-2.8441	-1.42745
H	-5.79343	-3.17345	-2.65202
H	-4.40615	-2.1707	-2.20639
H	-4.45887	-3.85681	-1.71534
H	-4.34945	-0.62555	2.854076
H	-5.68484	-1.26466	3.819545
H	-4.34152	-2.31239	3.345672
H	-9.77381	-2.25463	1.657664
H	-9.77141	-1.27688	0.189267
H	-9.80209	-3.03466	0.070708

6. IR and Raman Spectroscopies

Quantum chemical calculations. For simulating the IR and Raman spectra, density-functional theory (DFT) calculations were performed on the isolated full molecular model shown in Figure S26 by means of the Gaussian09 code[2]. The B3LYP functional has been chosen together with the 6-31G(d,p) basis set, consistently with the extensive use of this method in the study of molecular graphene.[6]

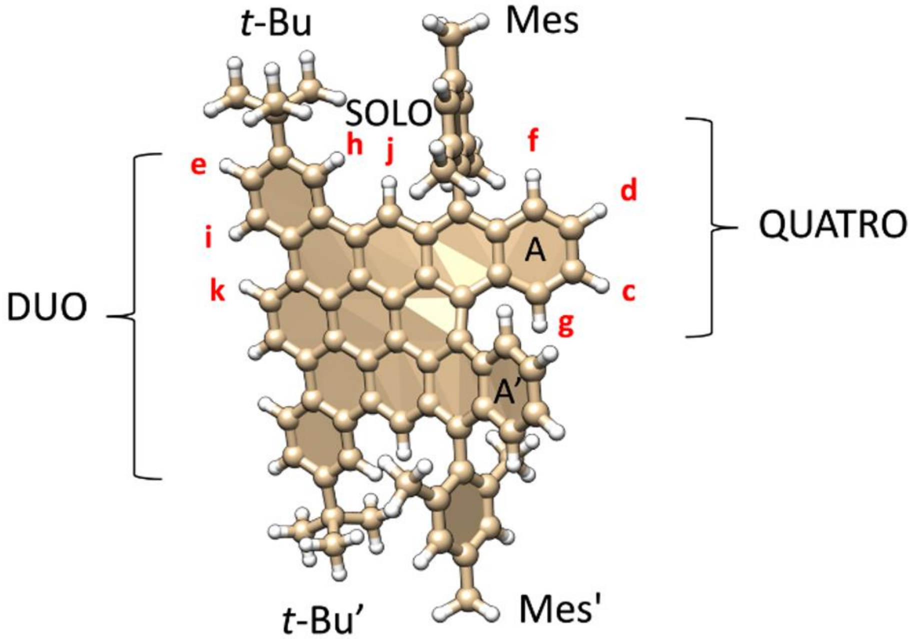

Figure S26. Equilibrium geometry of the molecular model of DBDNC **1** obtained at the B3LYP/6-31G(d,p) level. Labelled sites are referred to in the vibrational assignments of Tables S1, S3; primed sites are equivalent by symmetry. The labeling of hydrogens follows that of Figure 2 in the main text; equivalent hydrogens are not indicated.

Table S3. Assignment of the most intense peaks of the IR spectrum of DBDNC **1** reported in Figure 3c. Labels are defined in Figure 3c. Primed labels are equivalent by symmetry to their non-primed counterparts. The adopted frequency scaling factor is 0.98.

Experimental peak position (cm^{-1})	Peak position in the simulated spectrum (cm^{-1})	Frequency scaled position of the computed IR transitions (cm^{-1})	Description
661	660	660	QUATRO, collective CC stretching near A and A'
678	681	681	collective ring deformation
698	697	696	QUATRO, collective CC stretching near A and A'
703	714	713	collective CC stretching
740	748	748	QUATRO, ring deformation
757	762	762	QUATRO
769	773	769; 774	ring deformation in A and A', QUATRO
811	815	815	DUO (e-i, k-k', e'-i')
835	840	840	CC stretching in *t*-Bu and *t*-Bu', A and A'
850	854	855	SOLO in Mes and Mes'
873	862	862	SOLO (h, j, h', j')
881	893	892	SOLO (h, j, h', j')
908	907	907	ring deformation in A and A'
929	927	927	ring deformation in A and A'
956	960	956	CH bending in A and A'
973	971	970	collective ring deformation, CH bending in A and A'

Table S4. Graphical representation of the IR-active normal modes mentioned in the assignment of Table S1. Red and blue circles represent out-of-plane nuclear displacements with opposite phases, their magnitude being proportional to the radius of the circle. Green and blue segments indicate in-plane bond-stretching modes with opposite phases, their thickness being proportional to the maximum displacement.

Computed wavenumber (cm^{-1})	Scaled wavenumber (cm^{-1})	Graphical representation	IR intensity (km/mol)
673	660	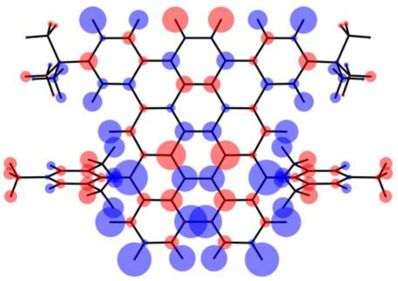	6
695	681	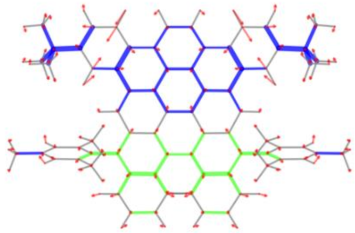	2
710	696	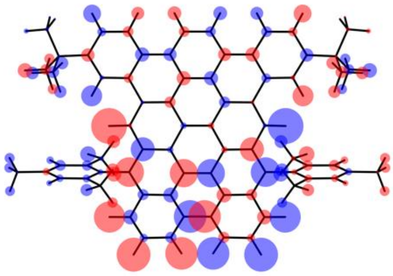	2
728	713	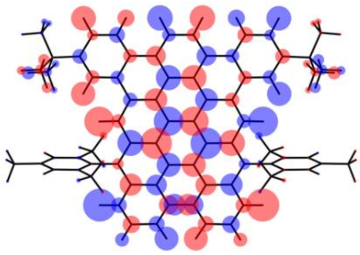	4
764	748	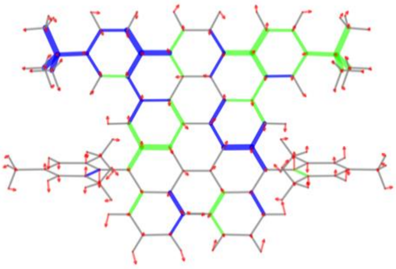	10

778	762	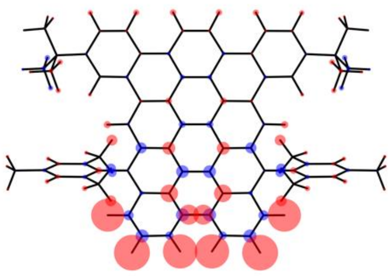	31
785	769	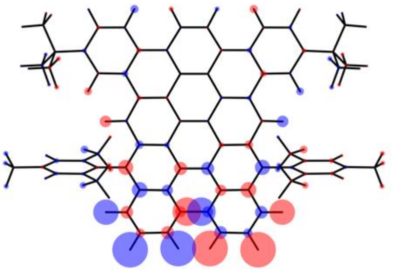	6
790	774	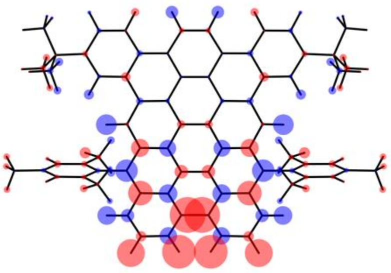	8
832	815	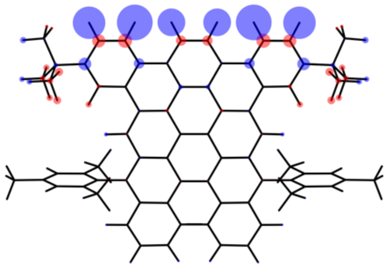	45
857	840	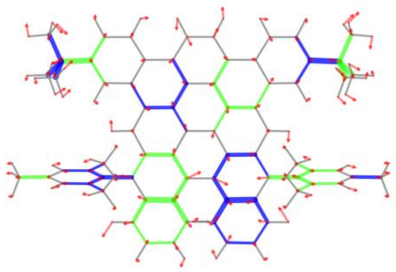	8

872	855	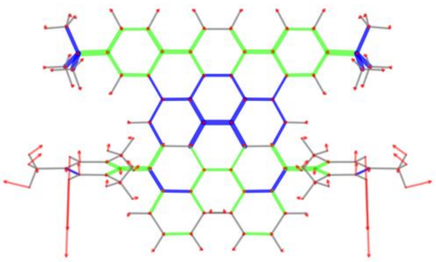	16
880	862	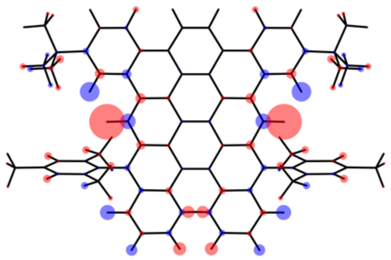	13
910	892	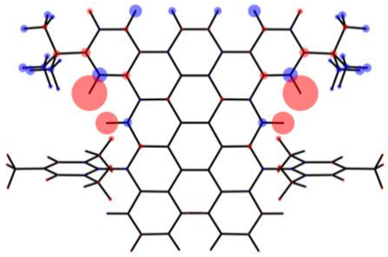	12
925	907	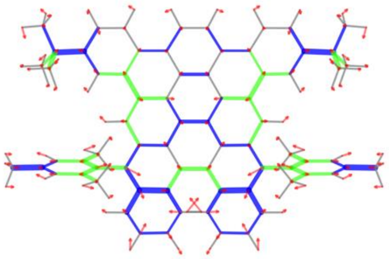	25
946	927	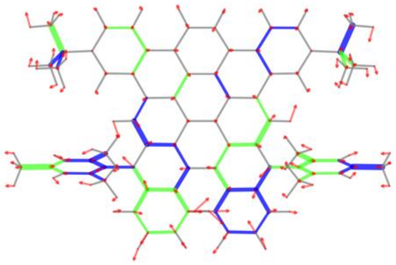	39
976	956	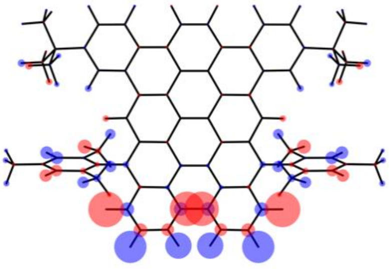	1

990	970	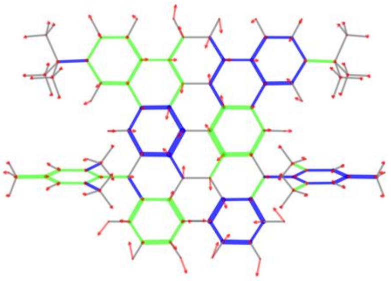	4

Table S5. Assignment of the most intense peaks of the Raman spectrum of DBDNC **1** reported in Figure 3d. Labels are defined in Figure 3d. Primed labels are symmetric equivalent to their non-primed counterparts.

**Experimental peak position (cm^{-1})**	**Peak position in the simulated spectrum (cm^{-1})**	**Frequency scaled position of the computed Raman transitions (cm^{-1})**	**Description**
1120	1130	1128	in-plane CH bending in e, i, k, e', i', k'
1174	1180	1179	in-plane CH bending in e, i, k, g, c, e', i', k', g', c'
1195	1202	1201	localized ring breathing, collective CH bending
1251	1258	1256	collective ring deformation and in-plane CH bending in e, i, k, e', i', k'
1266	1271	1272	localized ring breathing and in-plane CH bending in k, k'
1299	1300	1300	localized ring breathing and in-plane CH bending in j, j'
1322	1325	1325	ring breathing in k, k'
1349	1354	1353	collective CC stretching
1388	1385	1360	localized ring breathing
1517	1502	1501; 1507	localized G-like distortion. CH bending in Mes and Mes'; CH bending in *t*-Bu, *t*-Bu'
1565	1575	1576	G-like distortion
1602	1609	1608	ring stretching in k, k'

Table S6. Graphical representation of the Raman-active normal modes mentioned in the assignment of Table S3. Red and blue circles represent out-of-plane nuclear displacements with opposite phases, their magnitude being proportional to the radius of the circle. Green and blue segments indicate in-plane bond-stretching modes with opposite phases, their thickness being proportional to the maximum displacement.

**Computed wavenumber (cm^{-1})**	**Scaled wavenumber (cm^{-1})**	**Graphical representation / animation**	**Raman activity (Å^4/amu)**
1151	1128	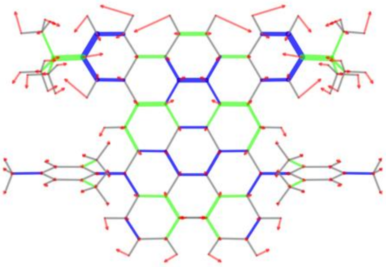	603

1203	1179	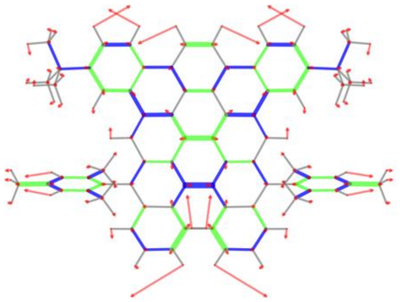	262
1225	1201	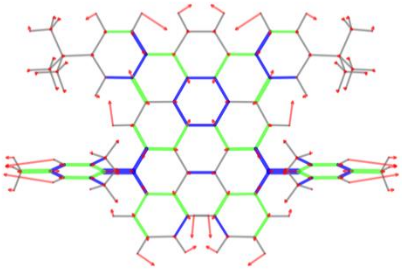	394
1282	1256	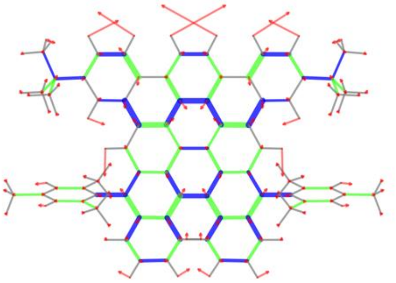	2172
1298	1272	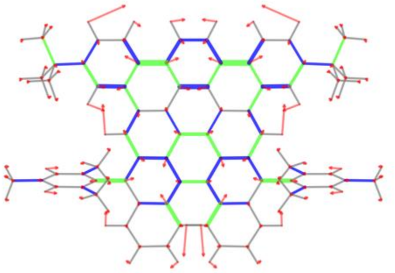	6556
1327	1300	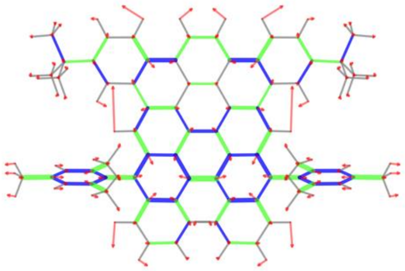	1898

1352	1325	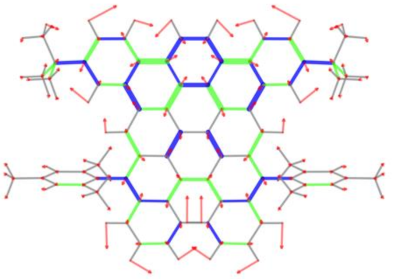	752
1381	1353	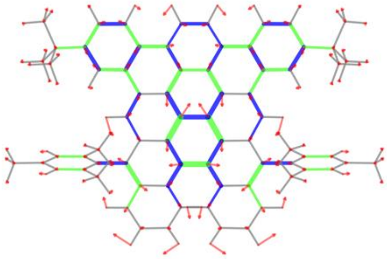	3616
1388	1360	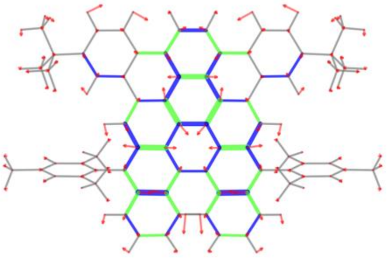	1794
1532	1501	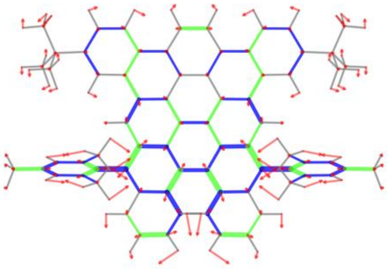	922
1538	1507	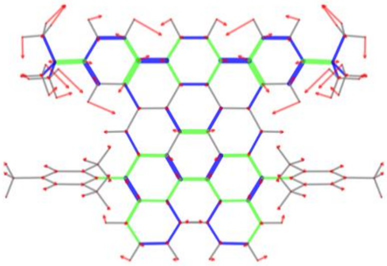	736

1608	1576	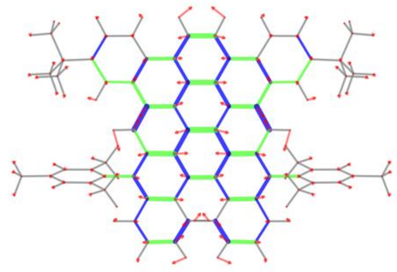	12272
1641	1608	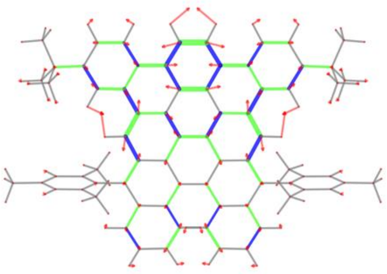	3477

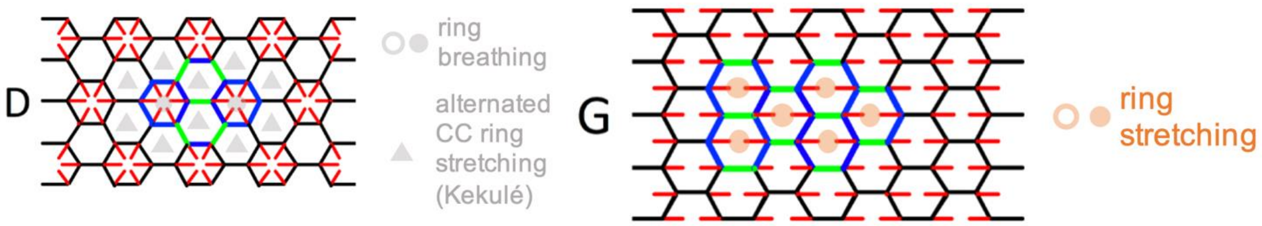

Figure S27. Representation of the D and G Raman modes. Green and blue segments indicate in-plane bond-stretching modes with opposite phases, their thickness being proportional to the maximum displacement. A key is also shown for the identification of the nuclear displacement patterns of the D and G modes (full and empty circles indicate opposite phase).

7. Electrochemical Properties

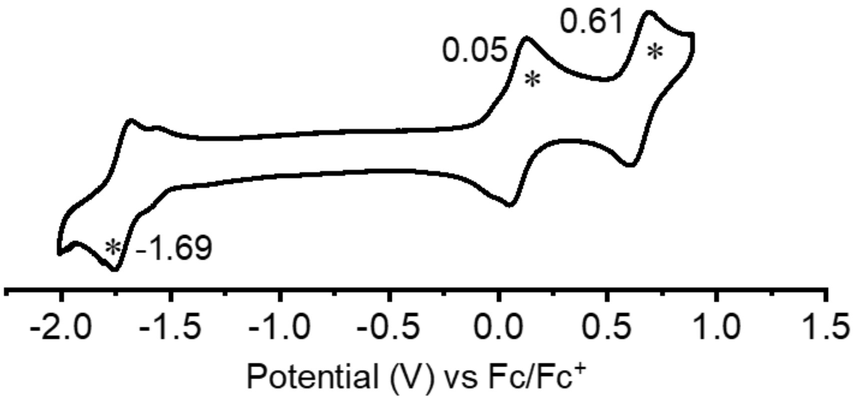

Figure S28. Cyclic voltammetry of DBDNC **1** in a solution of *n*-Bu_4NPF_6 (5×10^{-4} M) in CH_2Cl_2 with a scan rate of 50 mVs^{-1} at room temperature.

8. Photophysical Spectra

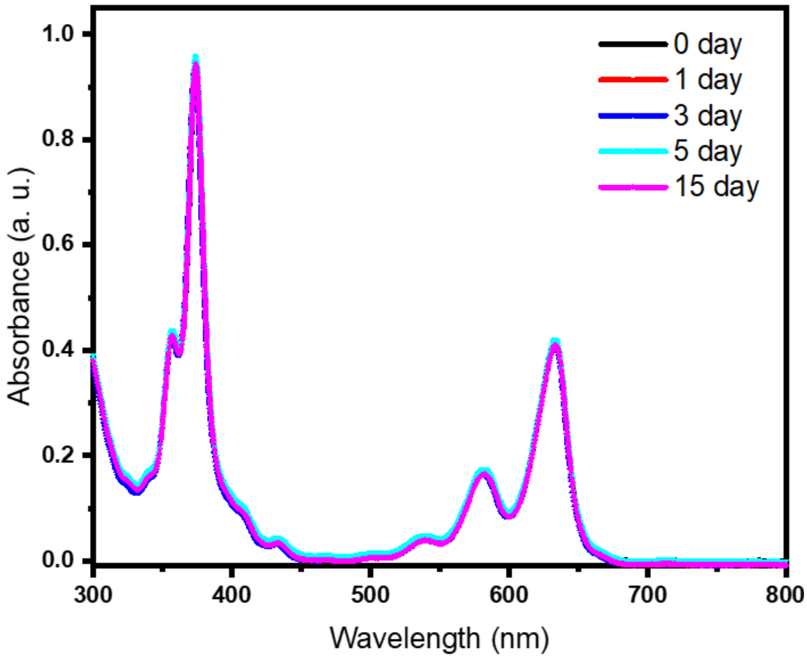

Figure S29. Solution stability of DBDNC **1** in toluene. The absorption spectra of freshly prepared solution and after storage under air in dark at room temperature for 1 day, 3 days, 5 days, and 15 days. The concentration of DBDNC **1** under test is 10^{-5} M.

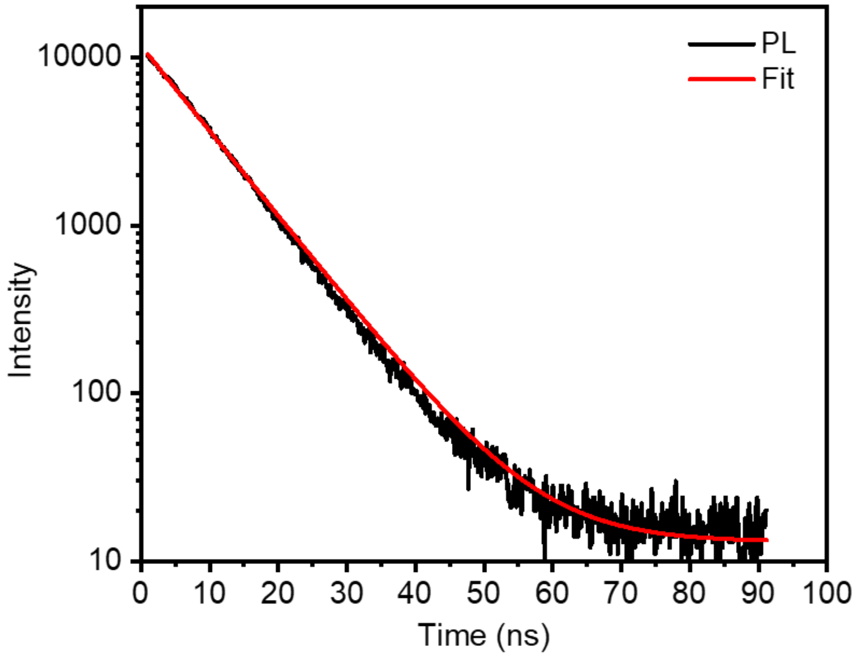

Figure S30. Emission lifetime decay curves for DBDNC **1** in toluene solution at room temperature and its mono-exponential fit. The lifetime τ was determined to be 8.5 ns. Concentration: 10^{-5}M. Excitation wavelength: 365nm. Integral wavelength range for emission: 540–795nm.

Table S7. Summary of photophysical data of DBDNC **1**.

	**λ_{abs}**[a] **[nm]**	**λ_{em}**[b] **[nm]**	**Φ_{PL}**[c]	**τ_{PL}**[d] **[ns]**	**E_{opt}**[d] **[eV]**	**HOMO/LUMO**[e] **[eV]**	**HOMO/LUMO**[f] **[eV]**
DBDNC **1**	373,582,633	643,698,772	0.53	8.7	1.94	-4.61/-2.61	-4.80/-3.17

[a] Measured in 10^{-5} M toluene solution. [b] Measured in 10^{-5} M toluene solution with excitation of 370 nm. [c] Absolute photoluminescence quantum yield measured in toluene solution. [d] PL decay lifetime measured at the emission peak in dilute toluene solution. [e] Optical energy gap (E_{opt}) was calculated according The optical energy gap was estimated from the interface of the absorption and emission spectra:1.94 eV: $E_{opt} = 1240/\lambda$. [e] HOMO/LUMO energy levels were calculated based on the density functional theory (DFT) at the B3LYP/6-311G(d,p) level. [f] HOMO and LUMO energy levels were calculated according to the following equations: HOMO = $-(4.8+E_{ox}^{onset})$; LUMO = $-(4.8+E_{red}^{onset})$, where E_{ox}^{onset} and E_{red}^{onset} onset referred to the onset potentials of the first oxidative and reductive redox waves, respectively.

9. Transient absorption

We employed an amplified Ti:Sapphire laser with 2 mJ output energy, 1 kHz repetition rate, a pulse width of ≈100 fs, and a central energy of 1.59 eV (800 nm). We used a pump wavelength of 630 nm, in resonant with the main $\pi \rightarrow \pi^*$ transition, with a spot diameter of 175 µm, a pump energy of 22 nJ, and a fluence of 73 µJ cm^{-2}. Such pump pulses were generated by using optical parameter amplification (OPA) in the visible range. As a probe pulse, we used a broadband white- light supercontinuum generated in a sapphire plate, extending from 430 to 800 nm. The pump–probe delay was set by means of a mechanical delay stage. DBDNC **1** was dissolved in toluene with a concentration of 0.1 mg mL^{-1}.

In a typical transient absorption experiment the pump pulse distributes excitation to the excite states, while the absorption spectrum of the pump-perturbed pulse is obtained via a probe pulse. The temporal delay between the pump and the probe pulses allows tracking out the temporal evolution of the excited states. In general, if one monitor the differential transient transmission of the probe as a function of the wavelength, it can be possible discriminating three kinds of signals. At a probe wavelength corresponding to the ground state absorption, the transmission of the probe is enhanced due to the pump-induced depletion of the ground state, and the positive signal is called photo-bleaching ($\Delta T/T > 0$, PB). On the other hand, $\Delta T/T$ is negative when the probe transmission is attenuated by excited states absorption events, and the relative signals is called excited state absorption ($\Delta T/T < 0$, ESA). The probe pulse can also stimulate the emission of photons from the excited states, leading to a perceived increase of the transmission at the stimulated emission wavelengths (SE, $\Delta T/T > 0$). Data were fitted by using a double or three-exponential model by using the software OriginPro 2022. In particular, in DBDNC **1** the SE relaxation can be modelled via two-exponentials (3 ps and 6 ns, due to internal conversion and radiative relaxation from S1), while in DBOV-Mes-C12 we have an additional fitting component (170 ps) that can be attributed to charge transfer state in aggregates. In addition, the long component (relaxation from S1) is sensibly shorter in DBOV than in DBDNC **1** (2 ns).

10. Amplified spontaneous emission

The samples manufactured for the amplified spontaneous emission (ASE) characterization consisted of polystyrene (PS) thin films doped with 1 wt% of DBDNC **1**, which were fabricated by spin-coating a toluene solution over fused silica substrates. The film thickness (h_f = 530 nm) was determined by using a non-invasive interferometric technique.[7] The samples constitute waveguides with optimal conditions for light amplification, i.e., these only support fundamental transversal electric (TE_0) and magnetic (TM_0) modes with high confinement factor ($\Gamma \approx 90$ %).[8-9] The films ASE was characterized under ns-pulsed excitation by pumping the sample with the third harmonic output of a Nd:YAG laser (10 Hz, λ_{pump} = 355 nm). The laser bean was projected over the sample as a narrow stripe (0.5 mm by 3.5 mm) by means of a cylindrical lens and a slit and the pump intensity was modulated with neutral density filters. The light emitted by the sample was collected from the film's edge by means of an optical fiber attached to an USB2000 spectrometer (Ocean Optics), where the emission was analyzed. The ASE threshold (E_{th}) was determined as the pump fluence at which the linewidth, defined as full width at half maximum (FWHM), reaches the average value between low and high pump fluence, which coincides with the slope change in the output intensity versus pump fluence representation.[10] Finally, the ASE photostability of the compound was tested under uninterrupted ns-pulsed excitation with a fluence twice the ASE threshold and the photostability half-live parameter ($\tau_{1/2}$) was defined to quantify the performance as the time or number of pump pulses needed for the output intensity to reach half its initial value.

Table S8. Amplified spontaneous emission parameters of DBDNC **1** dispersed at 1 wt% in a polystyrene thin film. For comparative purposes, data for films containing 1 wt% of other nanographenes are included.

Nanographene	$\lambda_{pump}{}^{a}$ [nm]	$\tau_{pump}{}^{b}$ [ns]	$\alpha_{pump}{}^{c}$ [×10^3 cm^{-1}]	$\lambda_{ASE}{}^{d}$ [nm]	FWHM^e [nm]	$E_{pump}{}^{f}$ [mJ cm^{-2}]	$\tau_{1/2}{}^{g}$ [pp]	reference
DBDNC	355	5.7	1.3	655	5	13	400	
				700	8	5.3	400	
FZ3	613	4.5	0.56	685	3	60	~10^5	11
				739	6	4.4	~10^5	11
PP-Ar	646	4.6	0.52	726	6	60	0.4×10^5	12
				787	9	1.5	10×10^5	12

^{a}Pump wavelength; ^{b}Pump pulse width at λ_{pump}; ^{c}Absorption coefficient at λ_{pump} (error ~ 2%); ^{d}Amplified spontaneous emission (ASE) wavelength (± 1 nm); ^{e}ASE linewidth, defined as the full width at half maximum for a pump fluence well above the threshold (± 1 nm); ^{f}ASE threshold determined under ns-pulsed excitation (10 Hz) expressed as an energy density per pulse (error ~ 10%); ^{g}ASE photostability half-life under uninterrupted pulsed pump with a fluence twice E_{th} expressed in pump pulses (error ~ 20%).

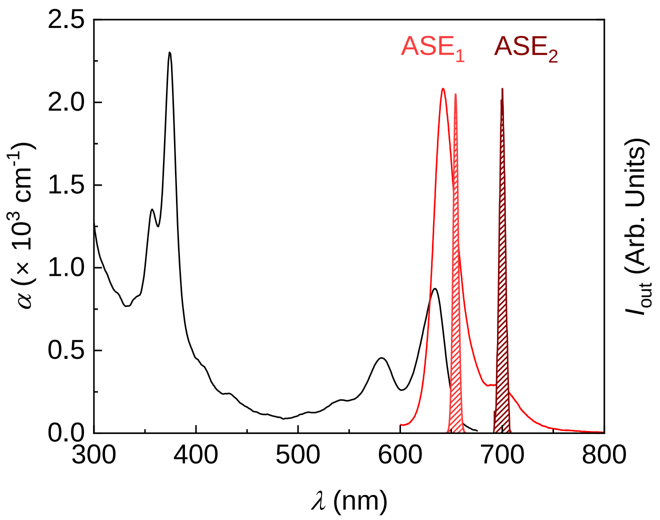

Figure S31. Absorption coefficient (black solid line, left axis), PL (red solid line, right axis) and ASE spectra (shaded area, right axis) of a polystyrene thin films doped with 1 wt% DBDNC **1** deposited over fused silica substrates.

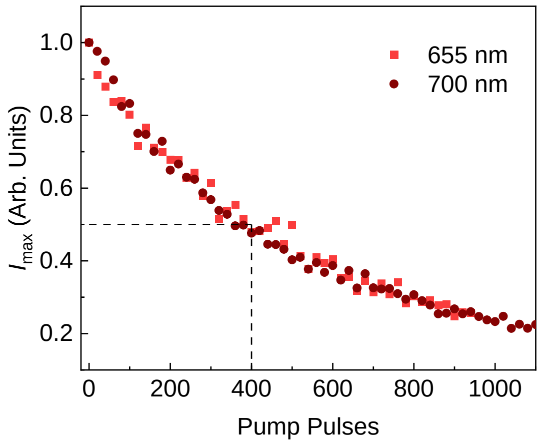

Figure S32. Evolution of the ASE peak output intensity (I_{max}) under uninterrupted ns-pulsed pump with a pump fluence twice the ASE threshold. Dashed lines are used to determine the photostability half-life ($\tau_{1/2}$ = 400 pump pulses).

References

1. Xu, X. S.; Di Giovannantonio, M.; Urgel, J. I.; Pignedoli, C. A.; Ruffieux, P.; Müllen, K.; Fasel, R.; Narita, A., On-surface activation of benzylic C-H bonds for the synthesis of pentagon-fused graphene nanoribbons. *Nano Res.* **2021,** *14* (12), 4754-4759.
2. Gaussian 09, R. D., Frisch, M. J.; Trucks, G. W.; Schlegel, H. B.; Scuseria, G. E.;; Robb, M. A. C., J. R.; Scalmani, G.; Barone, V.; Mennucci, B.; Petersson, G. A.;; Nakatsuji, H. C., M.; Li, X.; Hratchian, H. P.; Izmaylov, A. F.; Bloino, J.; Zheng, G.;; Sonnenberg, J. L. H., M.; Ehara, M.; Toyota, K.; Fukuda, R.; Hasegawa, J.; Ishida, M.;; Nakajima, T. H., Y.; Kitao, O.; Nakai, H.; Vreven, T.; Montgomery, Jr., J. A.; Peralta, J.; E.; Ogliaro, F. B., M.; Heyd, J. J.; Brothers, E.; Kudin, K. N.; Staroverov, V. N.;; Kobayashi, R. N., J.; Raghavachari, K.; Rendell, A.; Burant, J. C.; Iyengar, S. S.;; Tomasi, J. C., M.;Rega, N.; Millam, N. J.; Klene, M.; Knox, J. E.; Cross, J. B.; Bakken,; V.; Adamo, C. J., J.; Gomperts, R.; Stratmann, R. E.; Yazyev, O.; Austin, A. J.;; Cammi, R. P., C.; Ochterski, J. W.; Martin, R. L.; Morokuma, K.; Zakrzewski, V. G.;; Voth, G. A. S., P.; Dannenberg, J. J.; Dapprich, S.; Daniels, A. D.; Farkas, Ö.;Foresman, J. B.; Ortiz, J. V.; Cioslowski, J.; Fox, D. J. Gaussian, Inc., Wallingford CT, 2013.
3. Lu, T.; Chen, F. W., Multiwfn: A multifunctional wavefunction analyzer. *J. Comput. Chem.* **2012,** *33* (5), 580-592.
4. Geuenich, D.; Hess, K.; Köhler, F.; Herges, R., Anisotropy of the Induced Current Density (ACID), a General Method To Quantify and Visualize Electronic Delocalization. *Chem. Rev.* **2005,** *105* (10), 3758-3772.
5. Gershoni-Poranne, R.; Stanger, A., Magnetic criteria of aromaticity. *Chem. Soc. Rev.* **2015,** *44* (18), 6597-6615.
6. Wang, X.-Y.; Urgel, J. I.; Barin, G. B.; Eimre, K.; Di Giovannantonio, M.; Milani, A.; Tommasini, M.; Pignedoli, C. A.; Ruffieux, P.; Feng, X.; Fasel, R.; Müllen, K.; Narita, A., Bottom-Up Synthesis of Heteroatom-Doped Chiral Graphene Nanoribbons. *J. Am. Chem. Soc.* **2018,** *140* (29), 9104–9107.
7. Bonal, V.; Quintana, J. A.; Muñoz-Mármol, R.; Villalvilla, J. M.; Boj, P. G.; Díaz-García, M. A., Sub-400 nm film thickness determination from transmission spectra in organic distributed feedback lasers fabrication. *Thin Solid Films* **2019,** *692*, 137580.
8. Calzado, E. M.; Ramírez, M. G.; Boj, P. G.; García, M. A. D., Thickness dependence of amplified spontaneous emission in low-absorbing organic waveguides. *Appl. Opt.* **2012,** *51* (16), 3287-3293.
9. Lee, K.-H.; Thomas, K.; Gocalinska, A.; Manganaro, M.; Pelucchi, E.; Peters, F. H.; Corbett, B., SiNx-induced intermixing in AlInGaAs/InP quantum well through interdiffusion of group III atoms. *J. Appl. Phys.* **2012,** *112* (9), 093109.
10. Milanese, S.; De Giorgi, M. L.; Anni, M., Determination of the Best Empiric Method to Quantify the Amplified Spontaneous Emission Threshold in Polymeric Active Waveguides. *Molecules* **2020,** *25* (13), 2992.
11. Muñoz-Mármol, R.; Bonal, V.; Paternò, G. M.; Ross, A. M.; Boj, P. G.; Villalvilla, J. M.; Quintana, J. A.; Scotognella, F.; D'Andrea, C.; Sardar, S., Dual amplified spontaneous emission and lasing from nanographene films. *Nanomaterials* **2020,** *10* (8), 1525.
12. Muñoz-Mármol, R.; Gordillo, F.; Bonal, V.; Villalvilla, J. M.; Boj, P. G.; Quintana, J. A.; Ross, A. M.; Paternò, G. M.; Scotognella, F.; Lanzani, G.; Derradji, A.; Sancho-García, J. C.; Gu, Y.; Wu, J.; Casado, J.; Díaz-García, M. A., Near-Infrared Lasing in Four-Zigzag Edged Nanographenes by 1D versus 2D Electronic π-Conjugation. *Adv. Funct. Mater.* **2021,** *31* (41), 2105073.
